# Association of white matter hyperintensities with cognitive decline and neurodegeneration

**DOI:** 10.3389/fnagi.2024.1412735

**Published:** 2024-09-12

**Authors:** Tao-Ran Li, Bai-Le Li, Xin-Ran Xu, Jin Zhong, Tai-Shan Wang, Feng-Qi Liu, the Alzheimer’s Disease Neuroimaging Initiative

**Affiliations:** ^1^Department of Neurology, The First Affiliated Hospital of Nanjing Medical University, Jiangsu Province Hospital, Nanjing, China; ^2^Department of Hematology, The First Affiliated Hospital of Nanjing Medical University, Jiangsu Province Hospital, Nanjing, China; ^3^Beijing Pediatric Research Institute, Beijing Children's Hospital, Capital Medical University, National Center for Children’s Health, Beijing, China; ^4^Department of Neurology, Yangzhou Friendship Hospital, Yangzhou, China

**Keywords:** Alzheimer’s disease, WMH, cerebral small vessel disease, Aβ, cognition, neurodegeneration

## Abstract

**Background:**

The relationship between white matter hyperintensities (WMH) and the core features of Alzheimer’s disease (AD) remains controversial. Further, due to the prevalence of co-pathologies, the precise role of WMH in cognition and neurodegeneration also remains uncertain.

**Methods:**

Herein, we analyzed 1803 participants with available WMH volume data, extracted from the ADNI database, including 756 cognitively normal controls, 783 patients with mild cognitive impairment (MCI), and 264 patients with dementia. Participants were grouped according to cerebrospinal fluid (CSF) pathology (A/T profile) severity. Linear regression analysis was applied to evaluate the factors associated with WMH volume. Modeled by linear mixed-effects, the increase rates (Δ) of the WMH volume, cognition, and typical neurodegenerative markers were assessed. The predictive effectiveness of WMH volume was subsequently tested using Cox regression analysis, and the relationship between WMH/ΔWMH and other indicators such as cognition was explored through linear regression analyses. Furthermore, we explored the interrelationship among amyloid-β deposition, cognition, and WMH using mediation analysis.

**Results:**

Higher WMH volume was associated with older age, lower CSF amyloid-β levels, hypertension, and smoking history (all *p* ≤ 0.001), as well as cognitive status (MCI, *p* < 0.001; dementia, *p* = 0.008), but not with CSF tau levels. These results were further verified in any clinical stage, except hypertension and smoking history in the dementia stage. Although WMH could not predict dementia conversion, its increased levels at baseline were associated with a worse cognitive performance and a more rapid memory decline. Longitudinal analyses showed that baseline dementia and positive amyloid-β status were associated with a greater accrual of WMH volume, and a higher ΔWMH was also correlated with a faster cognitive decline. In contrast, except entorhinal cortex thickness, the WMH volume was not found to be associated with any other neurodegenerative markers. To a lesser extent, WMH mediates the relationship between amyloid-β and cognition.

**Conclusion:**

WMH are non-specific lesions that are associated with amyloid-β deposition, cognitive status, and a variety of vascular risk factors. Despite evidence indicating only a weak relationship with neurodegeneration, early intervention to reduce WMH lesions remains a high priority for preserving cognitive function in the elderly.

## Introduction

Alzheimer’s disease (AD) is a leading cause of dementia and one of the major causes of disability, dependency, and even death among older people worldwide. However, the disease still lacks significantly effective disease-modifying therapy methods and cannot be cured (Long and Holtzman, 2019; Zheng and Wang, 2024). At present, AD is considered a distinctive entity, and is defined pathologically by the presence of the following specific neuropathological profiles: extracellular deposition of amyloid-β (Aβ) and intraneuronal presence of aggregated hyperphosphorylated tau proteins (Jack et al., 2018; Long and Holtzman, 2019; Zheng and Wang, 2024). In addition to these core features, vascular pathologies are important and should not be disregarded. According to several postmortem investigations, dementia with pure AD pathologies is uncommon, with the majority of patients exhibiting mixed brain pathological alterations (Schneider et al., 2007; Grinberg et al., 2013; Toledo et al., 2013). By employing data-driven analytical techniques, several researchers have identified vascular dysregulation as the earliest and strongest pathological biomarker of late-onset AD (Iturria-Medina et al., 2016). Recently, our and other research groups have outlined the relationship between AD and cerebrovascular injury (Love and Miners, 2016; Klohs, 2019; Cortes-Canteli and Iadecola, 2020; Li and Liu, 2022; Rost et al., 2022). Given the lack of effective therapies, determining the correlation between cerebrovascular lesions and AD could potentially guide interventions tailored to specific risks, and preserve cognitive function in the elderly population.

White matter hyperintensities (WMH), a classical imaging hallmark of cerebral small vessel disease (CSVD), are visualized as elevated intensities on T2-weighted or fluid-attenuated inversion recovery (FLAIR) magnetic resonance imaging (MRI), and their importance has been reemphasized in the latest consensus guidelines (STRIVE-2) (Duering et al., 2023). Similar to macroscopic infarcts, the contribution of cerebral microangiopathy to dementia is also substantial (Troncoso et al., 2008). However, it seems inappropriate to regard WMH purely as vascular issues (Garnier-Crussard et al., 2023). Notably, a recent investigation of autosomal dominant inherited AD revealed that increased WMH volume is an early imaging feature of preclinical AD (Lee et al., 2016). In terms of ordering, WMH was visible following amyloid and tau pathologies and prior to symptom onset, with a volume accumulation that may be due to alterations in these indicators (Lee et al., 2016; Luo et al., 2023). Whether amyloid and tau pathologies are linked to the occurrence of WMH is controversial. While some studies have found an associated between higher amyloid burden and larger WMH volumes in patients with dementia (van Westen et al., 2016; Walsh et al., 2020; van Waalwijk van Doorn et al., 2021), mild cognitive impairment (MCI) (Marnane et al., 2016; Wei et al., 2019; Walsh et al., 2020), and cognitively normal controls (NCs) (Kester et al., 2014; Marnane et al., 2016; Osborn et al., 2018; Graff-Radford et al., 2019; Wei et al., 2019; Walsh et al., 2020), other studies did not detect any such a relationship (Hedden et al., 2012; Marchant et al., 2013; Park et al., 2014; Luo et al., 2023; Ortner et al., 2023; Ying et al., 2023). Mixed results have also been obtained from different meta-analyses (Roseborough et al., 2017; Liu et al., 2018; Twait et al., 2023). In contrast, most studies assessing the association between WMH and tau biomarkers have failed to demonstrate any positive associations at any clinical stage (Osborn et al., 2018; Wei et al., 2019; Walsh et al., 2020; van Waalwijk van Doorn et al., 2021; Garnier-Crussard et al., 2023; Ortner et al., 2023), while only some studies on imaging pathology have offered positive results (Erten-Lyons et al., 2013; McAleese et al., 2015). It should further be emphasized that age effects may account for some of these cross-sectional associations (Luo et al., 2023). Further evaluation of these interactions is required to understand the role of WMH in the internal pathology and extrinsic phenotype of AD.

The connection between WMH, cognitive decline, and dementia has been well established in several large meta-analyses (Debette and Markus, 2010; Debette et al., 2019; Hu et al., 2021). Further, cross-sectional studies have suggested that WMH are more prevalent in patients with dementia (Yoshita et al., 2006; Gordon et al., 2015), and are associated with cognitive function across all major domains (van den Berg et al., 2018). However, previous studies have predominantly used clinical criteria for AD diagnosis without biomarkers, which can lead to misdiagnosis. The clinical phenotype involves a concentrated expression of intrinsic pathological changes (Li et al., 2022b). Thus, another caveat is that determining the contribution of WMH to cognitive dysfunction may be confounded by the core features of AD. Concurrent cerebrovascular lesions lower the threshold for the symptomatic manifestations of AD (Cortes-Canteli and Iadecola, 2020; Rost et al., 2022); however, whether the effects of WMH and AD pathologies on cognition are additive or synergistic remains unclear.

Herein, based on a large sample of well-characterized participants, we aimed to (1) explore the relationships between WMH and AD cerebrospinal fluid (CSF) core biomarkers along the cognitive continuum; (2) clarify the association between WMH and cognitive function among non-dementia participants who have the greatest therapeutic potential (Long and Holtzman, 2019; Zheng and Wang, 2024); (3) reveal the interactions among AD pathologies, WMH, and cognition; and (4) evaluate the relationship between WMH and neurodegeneration.

## Materials and methods

### Participants

Data used in the preparation of this article were obtained from the Alzheimer’s Disease Neuroimaging Initiative (ADNI) database (adni.loni.usc.edu). The ADNI was launched in 2003 as a public-private partnership, led by Principal Investigator Michael W. Weiner, MD. The primary goal of ADNI has been to test whether serial MRI, positron emission tomography (PET), other biological markers, and clinical and neuropsychological assessment can be combined to measure the progression of MCI and early AD. For up-to-date information, see http://www.adni-info.org.

**Figure 1:**
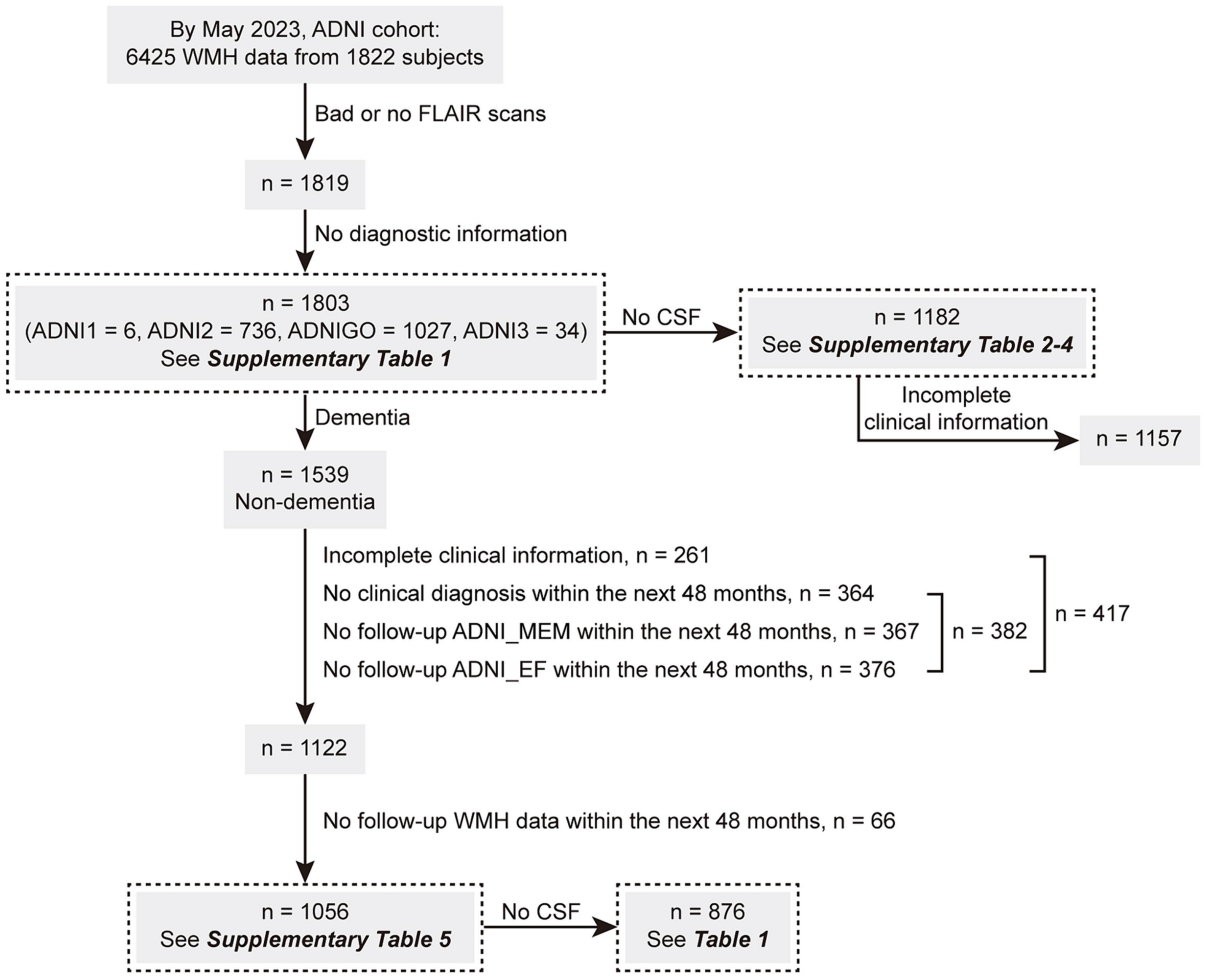
The screening flowchart of participants in this study. ADNI, Alzheimer’s Disease Neuroimaging Initiative; CSF, cerebrospinal fluid; MEM, memory sub-domain; EF, executive function sub-domain; WMH, white matter hyperintensity.

As of May 2023, this study enrolled 1803 individuals with available baseline WMH volume data. The ADNI database classified these individuals clinically as NCs (*n* = 756; Mini-Mental State Examination [MMSE] score: ≥ 24, Clinical Dementia Rating Scale [CDR] score: 0), patients with MCI (*n* = 783; MMSE score: ≥ 24, CDR score: 0.5, and objective memory loss measured using education-adjusted scores on delayed recall of logical memory), or patients with AD dementia (*n* = 264) following predefined criteria (McKhann et al., 1984). Among them, 1,182 individuals had available CSF data. On the other hand, 1,056 of the above 1,539 non-dementia participants (68.6%) had complete demographic information and underwent one or more clinical follow-ups and WMH reexaminations within the next 48 months. Among this cohort, CSF data were available for 876 individuals. Figure 1 illustrates the screening process, and the clinical information of the enrolled participants is presented in Supplementary Tables S1–S5 and Table 1.

**Table 1:** Baseline participant characteristics.

	NC (*N* = 391)		MCI (*N* = 485)	
	Mean ± SD (or %)	*n*	Mean ± SD (or %)	*n*
Age at baseline	73.4 ± 6.44	391	71.8 ± 7.56^#^	485
Male	162 (41.4%)	391	271 (55.9%)^***^	485
Education	16.7 ± 2.39	391	16.2 ± 2.64^**^	485
*APOE* ε4−/−	266 (68.0%)	391	254 (52.4%)^***^	485
*APOE* ε4+/−	112 (28.6%)	391	177 (36.5%)^**^	485
*APOE* ε4+/+	13 (3.3%)	391	54 (11.1%)^***^	485
**Past medical histories**				
Hypertension	179 (45.9%)	390	237 (48.9%)^#^	485
Diabetes	47 (12.1%)	390	55 (11.3%)^#^	485
Hyperlipidemia	204 (52.3%)	390	264 (54.4%)^#^	485
Smoking	117 (30.0%)	390	170 (35.1%)^**^	485
Atrial fibrillation	17 (4.4%)	390	17 (3.5%)^#^	485
Coronary artery diseases	37 (9.5%)	390	55 (11.3%)^#^	485
Cerebrovascular diseases	18 (4.6%)	390	20 (4.1%)^#^	485
**Neuropsychological scales**				
MMSE	29.09 ± 1.142	391	28.06 ± 1.763^***^	485
MoCA	25.84 ± 2.584	389	23.22 ± 3.143^***^	484
ADAS-cog13	8.55 ± 4.401	388	14.92 ± 6.671^***^	483
Logical Memory	13.42 ± 3.212	391	7.03 ± 3.264^***^	484
ADNI_MEM	1.08 ± 0.594	391	0.34 ± 0.664^***^	485
ADNI_EF	0.97 ± 0.820	391	0.40 ± 0.883^***^	485
ADNI_LAN	0.88 ± 0.721	391	0.36 ± 0.761^***^	485
ADNI_*VS*	0.20 ± 0.634	391	−0.01 ± 0.734^***^	485
**CSF core biomarkers**				
Aβ_42_ (pg/mL)	1354.16 ± 662.193	391	1067.36 ± 565.004^***^	485
p-tau (pg/mL)	21.64 ± 9.452	391	26.46 ± 14.724^***^	485
t-tau (pg/mL)	237.93 ± 91.014	391	274.95 ± 129.263^***^	485
**CSF GAP43 (pg/mL)**	4952.72 ± 2712.091	235	5149.99 ± 2858.624^#^	396
**Neuroimaging**				
FDG (metaROI SUVR)	1.30 ± 0.054	252	1.27 ± 0.070^***^	476
B_HP volume (cm^3^)	6.69 ± 0.744	391	6.37 ± 0.921^***^	485
GM volume (cm^3^)	505.08 ± 47.354	391	496.86 ± 49.091^*^	485
Ent_thickness (mm)	3.57 ± 0.285	391	3.35 ± 0.464^***^	485
WMH volume (cm^3^)	5.20 ± 9.113	391	6.57 ± 8.744^*^	485
WMH volume (Logarithmization)^a^	0.38 ± 0.564	391	0.51 ± 0.561^***^	485
WMH volume (Logarithmization)^b^	−2.77 ± 0.564	391	−2.63 ± 0.558^***^	485

The analysis was performed in a subset of the included 1,803 participants, these individuals were non-dementia, had complete demographic information and received one or more clinical follow-up and WMH reexamination within the next 48 months, and had available CSF data (*n* = 876). Categorical and continuous measures are presented as numbers (%) or means ± standard deviations. The statistical analyses were conducted by chi-square tests for categorical variables and independent two-sample *t*-tests for continuous variables. *, < 0.05; **, < 0.01; ***, < 0.001; #, > 0.05. **Coronary artery diseases** include patients who have (or have not) undergone stent placement or bypass grafting. **Cerebrovascular diseases** refer to transient ischemic attack and ischemic stroke. **Logical memory** indicates the total number of story units that are recalled. **ADNI_MEM/EF/LAN/VS** refers to the composite measures of memory function/executive function/language function/visuospatial function derived from the ADNI database. **MetaROI** includes the left angular gyrus, right angular gyrus, bilateral posterior cingulate gyrus, left inferior temporal gyrus, and right inferior temporal gyrus, which are the most important hypometabolic regions indicative of pathological metabolic changes in patients with AD. **B_HP volume** indicates the total volume of bilateral hippocampus. **GM volume** indicates the cerebral gray matter volume. **Ent_thickness** indicates the average thickness of bilateral entorhinal cortices. **WMH volume (Logarithmization)** means natural log-transformed WMH (a) or log-transformed total intracranial volume-normalized WMH (b). NC, cognitively normal control; MCI, mild cognitive impairment; APOE, apolipoprotein E; MMSE, Mini-Mental State Examination; MoCA, Montreal Cognitive Assessment; ADAS-Cog 13, Alzheimer’s Disease Assessment Scale-Cognitive 13; ADNI, Alzheimer’s Disease Neuroimaging Initiative; MEM, memory sub-domain; EF, executive function sub-domain; LAN, language sub-domain; VSP, visuospatial sub-domain; CSF, cerebrospinal fluid; Aβ, β-amyloid; p-tau, phosphorylated tau; t-tau, total tau; GAP43, growth-associated protein-43; FDG, [18F]fluoro-2-deoxyglucose; ROI, region of interest; SUVR, standardized uptake value ratio; B, bilateral; HP, hippocampus; GM, gray matter; Ent, entorhinal cortex; WMH, white matter hyperintensity; AD, Alzheimer’s disease.

The included participants provided detailed clinical information, including demographic data, apolipoprotein E (APOE) status, history of chronic diseases, smoking history, and neuropsychological test results. The tests included the CDR, MMSE, Montreal Cognitive Assessment, 13-Item Alzheimer’s Disease Assessment Scale-Cognitive Subscale, and Logical Memory test. Composite scores for memory (MEM), executive function (EF), language, and visuospatial function derived from the ADNI were also obtained (Crane et al., 2012). The corresponding tables illustrate the available data.

### CSF biomarkers

AD core biomarkers, including the levels of CSF Aβ_42_, p-tau (phosphorylated at threonine 181), and t-tau, were measured using fully automated Roche Elecsys^®^ immunoassays, as described previously (Bittner et al., 2016). The enrolled participants were classified as having high brain Aβ loads (A+) or fibrillar tau (T+), according to *a priori* principles that utilized established cutoff values of <977 pg./mL for CSF Aβ_42_ and > 27 pg./mL for p-tau (Li et al., 2022a; Zhang et al., 2022). Growth-associated protein 43 (GAP43), a presynaptic protein indicative of nerve damage, was assayed using an in-house enzyme-linked immunoassay method that has been previously described in detail (Zhang et al., 2022).

Details regarding CSF collection and detection are available at http://adni.loni.usc.edu/.

### Neuroimaging acquisition and processing

WMH measurement was performed in the DeCarli Lab (UC-Davis), using a Bayesian approach for the segmentation of high-resolution 3D T1 and FLAIR sequences (Carmichael et al., 2012). In brief, images were processed to (1) exclude non-brain tissues, (2) spatially align, and (3) remove MRI field artifacts. The images were then warped to a standard template space in which the prior probability of WMH occurrence and the FLAIR signal characteristics of WMH were modeled at every location in the cerebral white matter. This prior information, together with the signal intensities of the FLAIR images in question, was used to identify WMH. To obtain structural indicators, including hippocampal volume, cerebral gray matter volume, and thickness of the entorhinal cortex, a region-of-interest (ROI)-based analysis was performed using FreeSurfer.^1^ The sum of the bilateral hippocampal volumes and the average bilateral thickness were used for subsequent analyses. For [^18^F]fluoro-2-deoxyglucose (^18^F-FDG) PET, the metaROI standardized uptake value ratio (SUVR) was calculated as representative of global glucose metabolism. The meta-ROI results from a set of predefined ROIs based on coordinates frequently cited in other AD-related FDG studies were used (Landau et al., 2011).

Details regarding imaging acquisition protocols and processing are also available online.^2^

### Statistical analysis

Basic participant characteristics are summarized as numbers (%) or means ± standard deviations for categorical and continuous variables, respectively. Chi-square tests were used for categorical variables, and one-way ANOVA followed by Tukey’s tests (or independent two-sample *t*-tests) were used for continuous variables. Notably, the WMH values were normalized by their respective total intracranial volumes (TIVs), and subsequently natural log-transformed to achieve a normal distribution because the values were skewed (Shapiro–Wilk test, *p* < 0.05). The natural log-transformed TIV-normalized WMH volume variable was used in subsequent analyses.

With reference to previous methods (Burt et al., 2019; Mattsson et al., 2019), we applied a linear mixed-effects model to assess how WMH volume changed over time across the different groups. Fixed effects included age, sex, educational level, and *APOE* ε4 status, TIV, baseline WMH volume, and a predefined group × time interaction, which, if statistically significant, indicated a difference of WMH change over time. The random effects included time, in which both the intercept and slope could vary. On the other hand, the change slope of WMH volume (ΔWMH), ADNI_MEM (ΔMemory), ADNI_EF (ΔEF), typical neurodegeneration markers such as hippocampal volume were estimated for subsequent analysis with the fixed effect of time. Spearman correlation analyses were further applied to evaluate the correlation between WMH volume and other indices, such as scores on typical neuropsychological scales, and the results were presented in the form of a heatmap. Furthermore, linear regression models were constructed to explore the factors associated with WMH volume, and to evaluate the relationships between WMH (or ΔWMH) and cognition or neurodegeneration. The covariates are displayed in the corresponding tables.

The predictive effectiveness of baseline WMH volume was verified by using Cox-proportional hazard regression models. The analyses included only NCs and MCI participants, with follow-up up to 48 months. The outcome of the model was time to dementia change. For each individual, time “0” was defined as the date of the baseline WMH assessment. For converters, the survival time was defined as the time from the baseline assessment to the diagnosis of dementia. The proportional hazards assumption was tested using the scaled *Schoenfeld* residuals.

Finally, mediation effect analysis was applied to explore the interrelationship among Aβ deposition, cognition, and WMH. The mediation was interpreted based on the estimated average direct effect (ADE), average causal mediation effect (ACME) and average total effect (ATE). The ADE represents the effect of the Aβ deposition on the outcome measure, and the ACME represents the effect of the Aβ deposition on the outcome through the mediator variable (WMH volume). The ATE is a summarisation of ADE and ACME. We calculated bias-corrected 95% confidence intervals for the size of the mediating effects using bootstrapping (*k* = 500 samples).

The significance threshold was set at *p* < 0.05 (corrected for multiple comparisons using Tukey’s test). The above-mentioned analyses were performed using GraphPad Prism (version 9.4.0) or R programming language (version 4.2.1; package “lmerTest,” “survival,” “mediation,” etc.).

## Results

### Basic characteristics

A total of 1803 participants, including 756 NCs, 783 patients with MCI, and 264 patients with dementia, were enrolled. As shown in Supplementary Table S1, the dementia group had a higher baseline age than the other two groups (75.4 ± 8.21 vs. 72.0 ± 7.25 [NC] and 72.4 ± 7.85 [MCI], both *p* < 0.001). Further, the NC group had a lower proportion of men and a slightly higher educational level than the other two groups (men: 41.3% vs. 55.1% [MCI] and 57.4% [dementia], both *p* < 0.001; education: 16.6 ± 2.42 vs. 16.2 ± 2.60 [MCI] and 15.6 ± 2.74 [dementia], *p* < 0.01 and 0.001, respectively). However, there were no differences in the frequency of chronic diseases such as hypertension and diabetes among the three groups, while patients with cognitive impairment were more likely to smoke (31.2% [MCI] and 32.3% [dementia] vs. 24.1% [NC], both *p* < 0.01). As expected, the MCI and dementia groups both showed worse performance scores in the neuropsychological tests and higher proportions of *APOE* ε4 carriers than the NC group (all *p* < 0.001); the AD CSF core biomarkers were also more altered in these two groups than in the NC group (all *p* < 0.001). Additionally, glucose metabolism, represented by the metaROI FDG SUVR, as well as several typical morphological parameters, including hippocampal volume, cerebral gray matter volume, and thickness of the entorhinal cortex, all gradually changed across the cognitive continuum (all *p* < 0.001).

A subset of the above cohort (*n* = 1,182) was further grouped by AD CSF core features. Details regarding group characteristics are presented in Supplementary Table S2 (by “A” status), Supplementary Table S3 (by “T” status), and Supplementary Table S4 (by both “A/T” profiles). Another subset included 1,056 non-dementia participants with complete demographic and follow-up information; of these, Supplementary Table S5 shows group characteristics and the numbers of participants with follow-up WMH/ADNI_MEM/ADNI_EF data within the next 48 months. Table 1 presents the clinical information of 876 individuals with CSF data, including 391 NCs and 485 patients with MCI. The differences between the two groups were consistent with those described above.

### Baseline WMH volume

**Figure 2:**
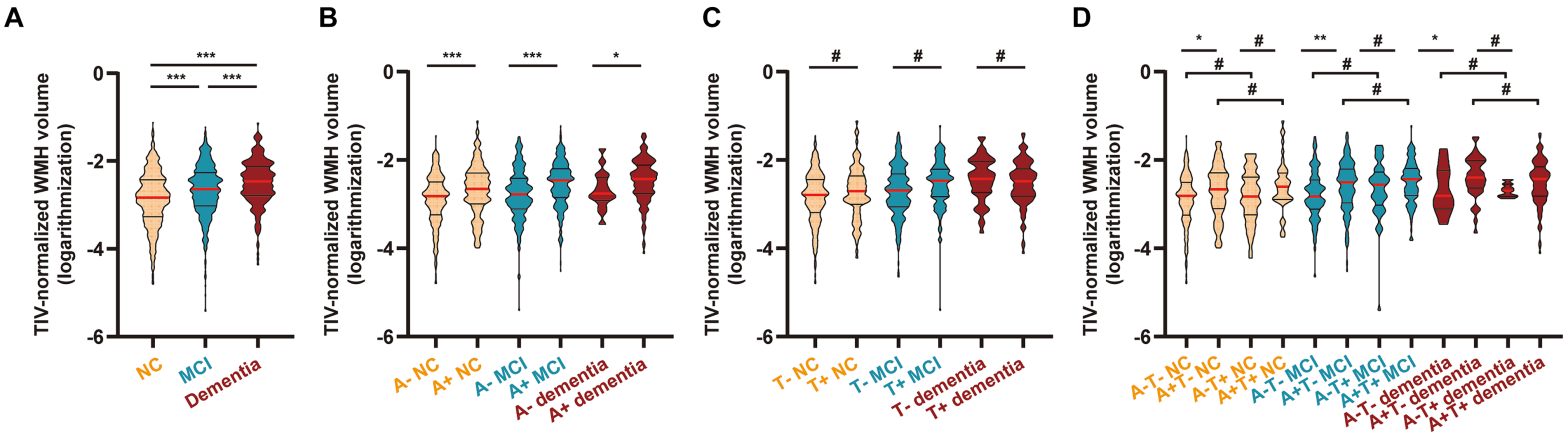
WMH volume in different diagnostic groups. **(A)** Participants were grouped by clinical diagnosis (*n* = 1803; see Supplementary Table S1). **(B)** Participants were grouped by clinical diagnosis and CSF-determined Aβ status (*n* = 1,182; see Supplementary Table S2). **(C)** Participants were grouped by clinical diagnosis and CSF-determined p-tau status (*n* = 1,182; see Supplementary Table S3). **(D)** Participants were grouped by clinical diagnosis and CSF-determined Aβ and p-tau statuses (*n* = 1,182; see Supplementary Table S4). According to a previous standard, we set the cutoff value at 977 pg./mL for Aβ and 27 pg./mL for p-tau to select participants with Aβ deposition (< 977 pg./mL; A+) and fibrillar tau (> 27 pg./mL; T+). The WMH volume was total intracranial volume-normalized and log-transformed (Supplementary Figure S2 shows the distribution of original WMH values and log-transformed WMH values). The results are presented in improved box charts; the red lines indicate the median values; the upper straight lines indicate the upper quartiles and the lower straight lines indicate the lower quartiles. Statistical analysis was conducted using one-way ANOVA followed by Tukey’s test (adjusted *p* value). Comparisons among groups: *, < 0.05; **, < 0.01; ***, < 0.001; #, > 0.05. WMH, white matter hyperintensity; NC, cognitively normal control; MCI, mild cognitive impairment; CSF, cerebrospinal fluid; Aβ, β-amyloid; p-tau, phosphorylated tau; ANOVA, analysis of variance.

As shown in Supplementary Table S1 and Figure 2A, WMH volume gradually increased across the cognitive continuum, with the lowest in the NC group, an increase in the MCI group (*p* < 0.001), and reaching the highest in the dementia group (vs. NC or MCI, both *p* < 0.001), suggesting that WMH volume may increase in relation to cognitive status. Correlation matrices displayed the Spearman correlation coefficients between WMH volume and scores on typical neuropsychology scales (Supplementary Figure S1; |R| = 0.151–0.332, *p* < 0.001), reinforcing the above suggestion. Next, the groups were further subdivided to explore the differences under different “A/T” profiles (Jack et al., 2018). After stratification of the clinically diagnosed groups additionally by Aβ status, the WMH volume was relatively higher in the A+ groups than in the corresponding A- groups (all *p* < 0.05; Supplementary Table S2 and Figure 2B). After stratification by p-tau status, no differences were observed between the paired groups (all *p* > 0.05; Supplementary Table S3 and Figure 2C). Following additional stratification by both Aβ and p-tau statuses, we observed that the transition of the p-tau condition did not influence the WMH volume under the same cognitive and Aβ statuses (all *p* > 0.05). In contrast, the A+ condition led to an increase in WMH volume in the T- NC groups (A− vs. A+, *p* < 0.05), T- MCI groups (A− vs. A+, *p* < 0.01), and T- dementia groups (A- vs. A+, *p* < 0.05). Aβ did not affect WMH in the T+ groups (A−T+ NC/MCI/dementia vs. corresponding A + T+ NC/MCI/dementia, all *p* > 0.05). The details are presented in Supplementary Table S4 and Figure 2D.

In addition to the cognitive status and Aβ levels, the above results indicated that there may be other factors influencing WMH volume. The regression analysis results further suggested that age (β: 0.029, *p* < 0.001), education level (β: −0.013, *p* = 0.038), hypertension (β: 0.120, *p* < 0.001), smoking history (β: 0.104, *p* = 0.001), cognitive impairment (β: 0.127 for MCI, *p* < 0.001; β: 0.136 for dementia, *p* = 0.008), and CSF Aβ_42_ levels (β: −0.0002, *p* < 0.001) were related to WMH. Meanwhile, sex, *APOE* ε4 status, CSF p-tau levels, and other chronic diseases (diabetes, atrial fibrillation, hyperlipidemia, cardiovascular and cerebrovascular diseases) were not associated with WMH (all *p* > 0.05; Table 2). In the subgroup analyses, age and CSF Aβ_42_ levels were still significant, while hypertension and smoking history were only significant in the non-dementia groups (Supplementary Table S6).

**Table 2:** Relationships between brain WMH volume and AD CSF core biomarkers.

	β	95% CI	SE	*p*
Intercept	−4.5634	−4.9623 ~ −4.1645	0.2033	**< 0.0001**
MCI group	0.1274	0.0617 ~ 0.1932	0.0335	**0.0001**
Dementia group	0.1355	0.0347 ~ 0.2362	0.0513	**0.0084**
Age	0.0288	0.0245 ~ 0.0330	0.0022	**< 0.0001**
Female	0.0556	−0.0058 ~ 0.1170	0.0313	0.0760
Education levels	−0.0126	−0.0245 ~ −0.0007	0.0061	**0.0375**
*APOE* ε4+/−	−0.0688	−0.1372 ~ 0.0001	0.0349	0.0586
*APOE* ε4+/+	−0.0330	−0.1487 ~ 0.0828	0.0590	0.5763
Hypertension	0.1199	0.0595 ~ 0.1802	0.0308	**0.0001**
Diabetes	0.0227	−0.0694 ~ 0.1149	0.0470	0.6285
Atrial fibrillation	0.0716	−0.0850 ~ 0.2281	0.0798	0.3699
Smoking	0.1038	0.0415 ~ 0.1660	0.0317	**0.0011**
Hyperlipidemia	0.0156	−0.0441 ~ 0.0752	0.0304	0.6093
Coronary artery diseases	−0.0129	−0.1087 ~ 0.0829	0.0488	0.7913
Cerebrovascular diseases	0.1178	−0.0344 ~ 0.2700	0.0776	0.1293
CSF Aβ_42_ levels	−0.0002	−0.0002 ~ −0.0001	< 0.0001	**< 0.0001**
CSF p-tau levels	0.0007	−0.0016 ~ 0.0031	0.0012	0.5395

The analysis was performed in a subset of the included 1,803 participants, these individuals had complete clinical information and available CSF data (*n* = 1,157; *n* = 467 for NC group, *n* = 526 for MCI group, and *n* = 164 for dementia group); compared with the participants in Supplementary Tables S2–S4, 25 participants were excluded due to incomplete clinical information. CSF core biomarkers, including Aβ_42_ and p-tau levels, plus diagnostic status, age, sex, education levels, APOE ε4 status, and comorbidities statuses were used as predictors of brain WMH volumes (total intracranial volume-normalized and log-transformed). CSF t-tau was not included due to its extremely high correlation with p-tau (*R* > 0.900, *p* < 0.001). Bold represents statistical significance. CI, confidence interval; SE, standard error; WMH, white matter hyperintensity; AD, Alzheimer’s disease; CSF, cerebrospinal fluid; MCI, mild cognitive impairment; APOE, apolipoprotein E; Aβ, β-amyloid; p-tau, phosphorylated tau; t-tau, total tau.

### Relationship between WMH and cognition

As shown in Table 3, higher WMH volume was associated with lower scores of ADNI_MEM (*p* = 0.040) and ADNI_EF (*p* = 0.002). The association was also significant for AD CSF core features. Furthermore, we found there were no interactive effects between WMH and CSF Aβ_42_ on both ADNI_MEM and ADNI_EF (*p* = 0.510 and 0.336, respectively; data not shown). When this interaction term was additionally included as a covariate, WMH was only significantly associated with ADNI_EF (*p* = 0.014), not ADNI_MEM (*p* = 0.129).

**Table 3:** Relationships between cognition and brain WMH volume.

	ADNI_MEM			ADNI_EF		
	*β*	95% CI	*p*	*β*	95% CI	*p*
MCI	−0.5755	−0.6495 ~ −0.5016	< 0.0001	−0.4809	−0.5886 ~ −0.3732	< 0.0001
Dementia	−1.5641	−1.6771 ~ −1.4513	< 0.0001	−1.4831	−1.6485 ~ −1.3183	< 0.0001
WMH	−0.0701	−0.1450 ~ −0.0048	0.0395	−0.1512	−0.2459 ~ −0.0566	0.0018
CSF Aβ_42_	0.000127	0.000066 ~ 0.000187	< 0.0001	0.000239	0.000151 ~ 0.000327	< 0.0001
CSF p-tau	−0.00892	−0.01156 ~ −0.00628	< 0.0001	−0.00539	−0.00924 ~ −0.00153	0.0062

The analysis was performed in a subset of the included 1,803 participants, these individuals had complete clinical information and available CSF data (*n* = 1,157; *n* = 467 for NC group, *n* = 526 for MCI group, and *n* = 164 for dementia group); compared with the participants in Supplementary Tables S2–S4, 25 participants were excluded due to incomplete clinical information. The WMH, CSF core biomarkers, including Aβ_42_ and p-tau levels, plus diagnostic status, age, sex, education levels, APOE ε4 status, and comorbidities statuses including hypertension and smoking history, were used as predictors of cognitive score. The WMH volume was total intracranial volume-normalized and log-transformed. CSF t-tau was not included due to its extremely high correlation with p-tau (*R* > 0.900, *p* < 0.001). ADNI, Alzheimer’s Disease Neuroimaging Initiative; MEM, memory sub-domain; EF, executive function sub-domain; CI, confidence interval; WMH, white matter hyperintensity; CSF, cerebrospinal fluid; MCI, mild cognitive impairment; APOE, apolipoprotein E; Aβ, β-amyloid; p-tau, phosphorylated tau; t-tau, total tau.

**Figure 3:**
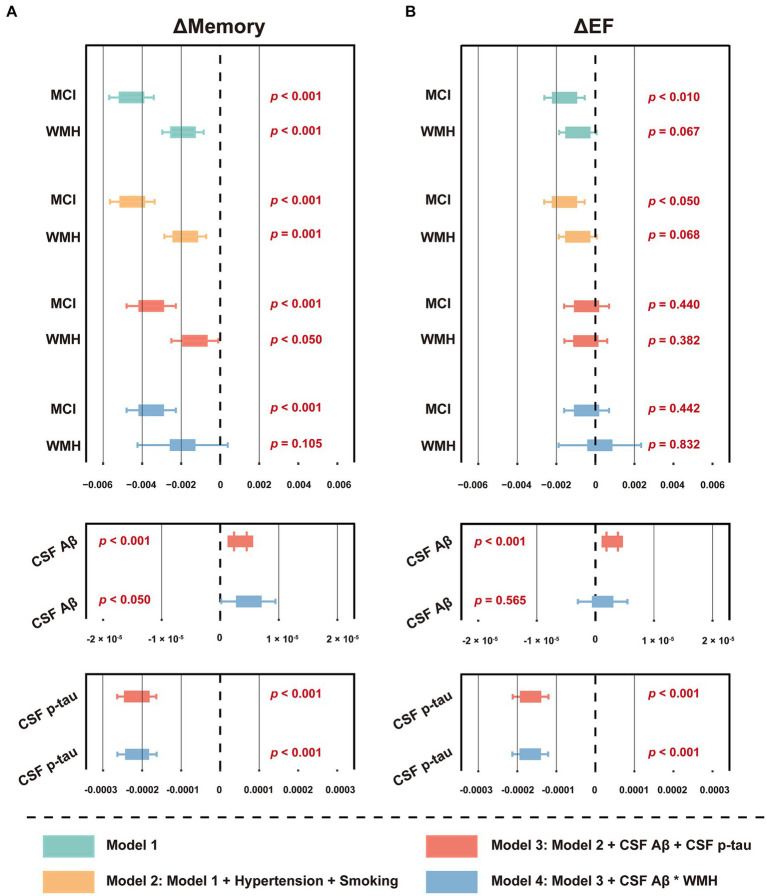
Forest plot of the association between baseline WMH volume and cognitive function slopes. **(A)** The association between baseline WMH volume and ΔMemory. **(B)** The association between baseline WMH volume and ΔEF. The cognitive function slopes were calculated by using linear mixed-effects models among non-dementia participants with at least one follow-up ADNI_MEM/EF score within the next 48 months (*n* = 1,056, see Supplementary Table S5). In model 1, baseline WMH volume, plus age, sex, education, *APOE* ε4 status, and cognitive status were used as predictors of cognitive function slopes. In model 2, hypertension and smoking status were used as additional predictors on the basis of model 1. In model 3, CSF core biomarkers, including Aβ_42_ and p-tau levels, were used as additional predictors on the basis of model 2. In model 4, the interaction term of CSF Aβ_42_ and WMH were used as additional predictors on the basis of model 3. The WMH volume was total intracranial volume-normalized and log-transformed. CSF t-tau was not included due to its extremely high correlation with p-tau (*R* > 0.900, *p* < 0.001). For effect estimates with exact 95% CI and statistical significance values, see Supplementary Table S9. MCI, mild cognitive impairment; WMH, white matter hyperintensity; CSF, cerebrospinal fluid; Aβ, β-amyloid; p-tau, phosphorylated tau; t-tau, total tau; APOE, apolipoprotein E; MEM, memory sub-domain; EF, executive function; ADNI, Alzheimer’s Disease Neuroimaging Initiative; CI, confidence interval.

Among the 876 participants summarized in Table 1, 119 (13.6%) developed dementia within the next 48 months, while the others developed MCI (4.3%), returned to a healthy status (4.8%), or remained stable (77.3%). Cox regression indicated that baseline WMH volume was not a risk factor for future dementia conversion (*p* = 0.840), unlike CSF Aβ_42_ or p-tau (both *p* < 0.001) (Supplementary Table S7). *Schoenfeld* residuals were computed to test proportional hazard assumption (Supplementary Table S8). However, linear regression analysis revealed that a higher WMH volume at baseline was associated with decreased levels in ΔMemory (Figure 3A and Supplementary Table S9), indicating that a larger baseline WMH volume was associated with a faster cognitive decline. Specifically, the effect of WMH on ΔMemory was not affected by demographic information, *APOE* ε4 status, baseline cognitive status, or previous disease status including hypertension and smoking (*p* < 0.001 and 0.010 for model 1 and model 2, respectively). In model 3, the CSF Aβ_42_ and p-tau levels were additionally included as covariates and the results were not altered (*p* < 0.05). In model 4, we observed no interactive effects between baseline WMH volume and CSF Aβ_42_ levels on ΔMemory (*p* = 0.611), while the WMH trended towards a significant increase the likelihood of lower ΔMemory (*p* = 0.105). In contrast, the WMH volume was not associated with ΔEF (Figure 3B and Supplementary Table S9). The association between AD CSF core features, especially CSF p-tau, and Δcognition was always significant.

### Longitudinal analyses

**Figure 4:**
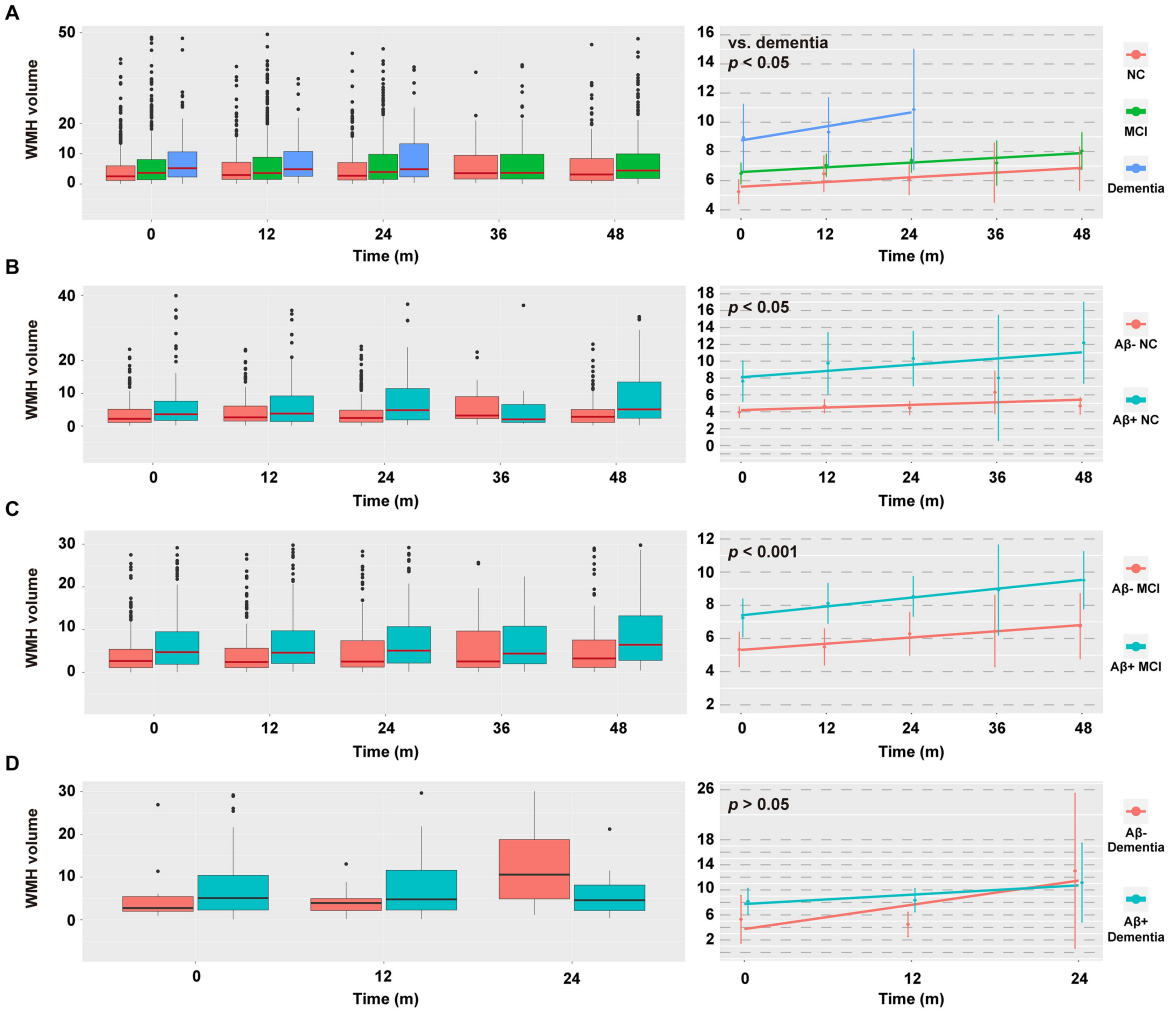
Data distribution and change in WMH volume within the next 48 months. **(A–D)** Data distribution of WMH volume (**left column**) and estimated means of WMH volume (**right column**) in different diagnostic groups. Raw values above 50/40/30/30 cm^3^ were discarded in A/B/C/D (**left column**) for the sake of aesthetics, with *n* = 24/15/55/13, respectively. Due to limited numbers, the follow-up WMH values cannot be predicted in the dementia group at the 36th and 48th month. The horizontal line in the middle of each box (**left column**) indicates the median, the top and bottom borders of the box mark the 75th and 25th percentiles. The modeled data (**right column**) show the mean 95% CIs for the predicted values using a linear mixed-effects model adjusted for age, sex, educational level, *APOE* ε4 status, total intracranial volume, and baseline WMH volume. *p* values indicate the group × time interaction effect. For effect estimates and statistical significance values, see Supplementary Tables S10, S11. NC, cognitively normal control; MCI, mild cognitive impairment; WMH, white matter hyperintensity; CSF, cerebrospinal fluid; Aβ, β-amyloid; APOE, apolipoprotein E.

Some participants had longitudinal WMH data at the 12th, 24th, 36th, and 48th months (Supplementary Tables S1, S5). The data distribution and changes in WMH volume are displayed in Figure 4. Group × time interactions were significant in participants with dementia (vs. NC or MCI, *p* < 0.05) and in participants with A+ and non-dementia profiles (A+ NC vs. A− NC, *p* < 0.05; A+ MCI vs. A− MCI, *p* < 0.001). Compared to their respective controls, the WMH volume of these participants increased faster over time (Figure 4; Supplementary Tables S10, S11). However, there were no differences of group × time interactions between the A+ dementia and A-dementia groups, or between the T+ groups and the corresponding T- groups (all *p* > 0.05; data not shown).

**Figure 5:**
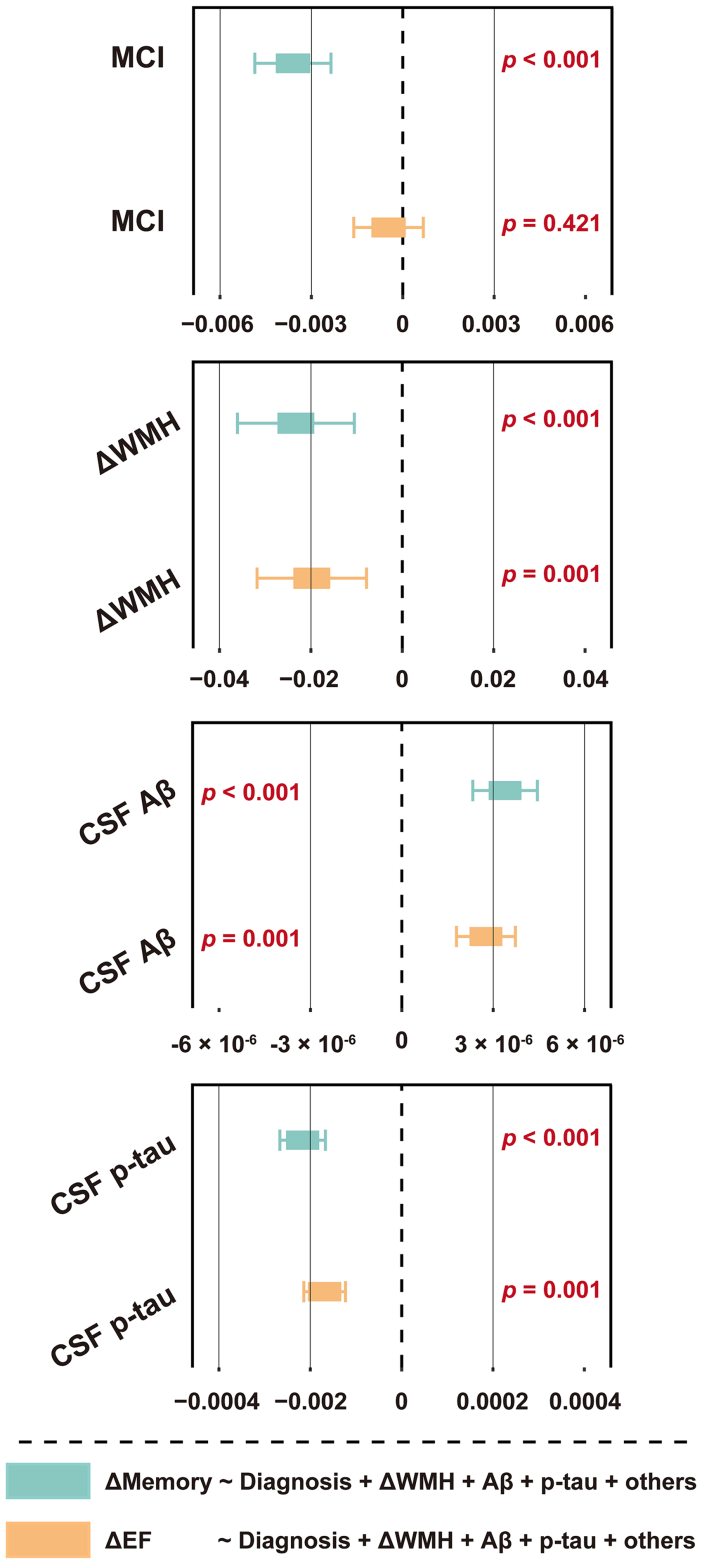
Forest plot of the association between ΔWMH volume and cognitive function slopes. The slopes (ΔWMH, ΔMemory and ΔEF) were calculated by using linear mixed-effects models among non-dementia participants with at least one follow-up WMH data, ADNI_MEM/EF score within the next 48 months (*n* = 1,056, see Supplementary Table S5). The ΔWMH volumes, plus diagnostic status, age, sex, education, *APOE* ε4 status, hypertension and smoking status, and CSF core biomarkers, including Aβ_42_ and p-tau levels were used as predictors of cognitive function slopes (*n* = 876, see Table 1). CSF t-tau was not included due to its extremely high correlation with p-tau (*R* > 0.900, *p* < 0.001). For effect estimates with exact 95% CI and statistical significance values, see Supplementary Table S12. MCI, mild cognitive impairment; WMH, white matter hyperintensity; CSF, cerebrospinal fluid; Aβ, β-amyloid; p-tau, phosphorylated tau; t-tau, total tau; APOE, apolipoprotein E; MEM, memory sub-domain; EF, executive function; ADNI, Alzheimer’s Disease Neuroimaging Initiative; CI, confidence interval.

Next, we explored the relationship between ΔWMH and Δcognition. As shown in Figure 5 and Supplementary Table S12, a higher ΔWMH was associated with decreased levels of ΔMemory and ΔEF (*p* < 0.001 and *p* = 0.001, respectively), indicating that a faster baseline WMH volume increase was associated with a more rapid cognitive decline. This association was not influenced by demographic information, *APOE* ε4 status, baseline cognitive status, or previous disease status including hypertension and smoking, and AD CSF core features.

### Mediation analyses

**Figure 6:**
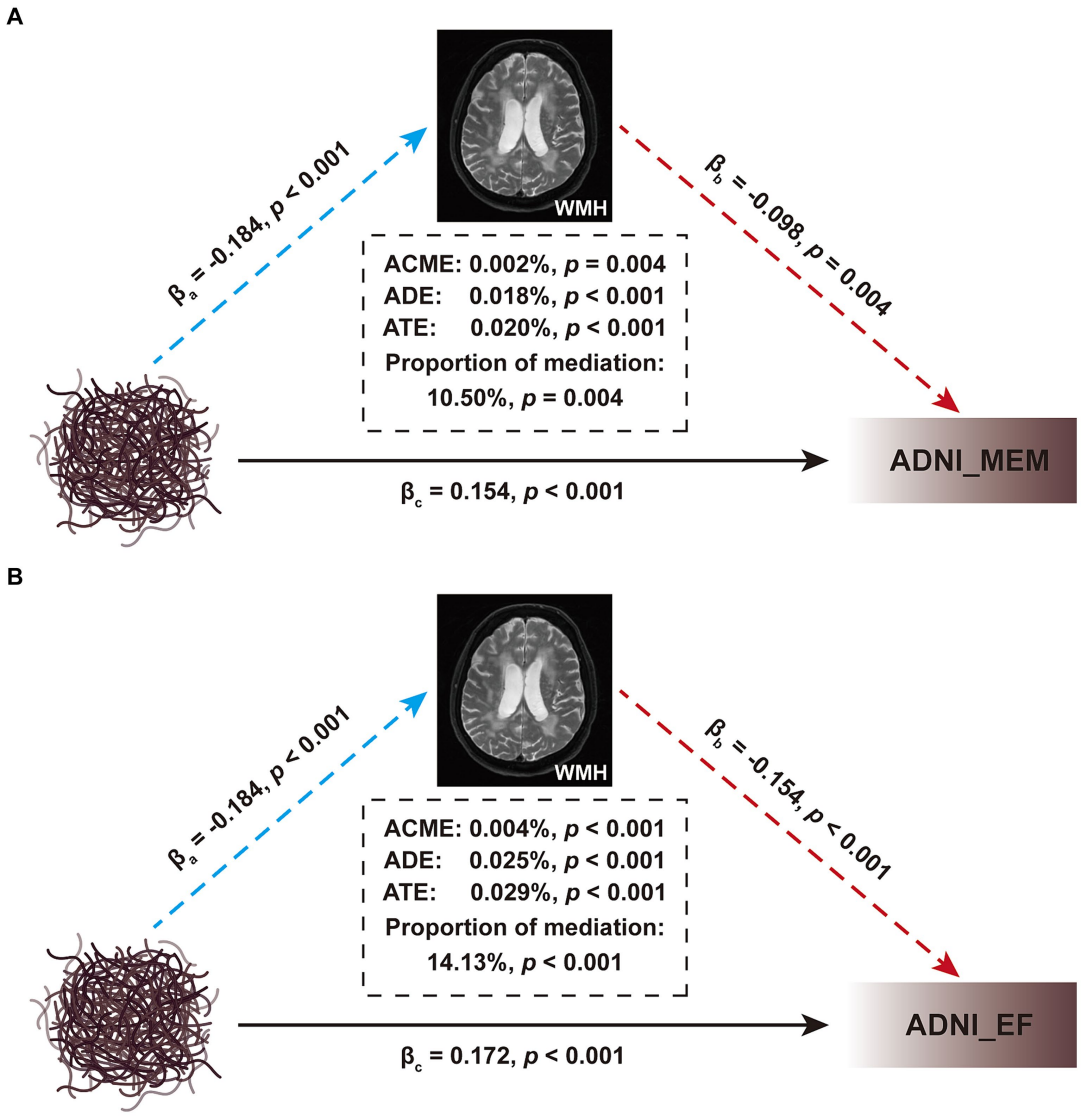
Mediation analyses of brain Aβ deposition on cognitive function. **(A)** The interrelationship among Aβ deposition, ADNI_MEM, and WMH. **(B)** The interrelationship among Aβ deposition, ADNI_EF, and WMH. Baseline WMH volume was used as the mediating variable; age, sex, educational level, and *APOE* ε4 status, were used as the covariates; the CSF Aβ levels were used as independent variable, and the ADNI_MEM or ADNI_EF score was used as dependent variable. All paths are presented in standardized regression coefficients (β). The WMH volume was total intracranial volume-normalized and log-transformed. The analysis was performed in 876 participants in Table 1. WMH, white matter hyperintensity; CSF, cerebrospinal fluid; Aβ, β-amyloid; APOE, apolipoprotein E; MEM, memory sub-domain; EF, executive function; ADNI, Alzheimer’s Disease Neuroimaging Initiative; ACME, average causal mediation effect; ADE, average direct effect; ATE, average total effect.

Considering the close relationship between CSF Aβ_42_ and WMH, as well as their impacts on cognitive function, we wondered whether WMH may mediate the relationship between brain Aβ deposition and cognition. Subsequent mediation analyses revealed both direct (i.e., ADE), WMH-mediated (i.e., ACME) and total effects of brain Aβ deposition on baseline cognitive performance (Figure 6). However, although significant, the mediation effects accounted for only 10.50% (*p* = 0.004) and 14.13% (*p* < 0.001) of the total effects in the MEM and EF cognitive domains, respectively. Further, we found that WMH also exerted significant mediating effects on the correlation between brain Aβ deposition and ΔMemory (proportion: 7.09%, *p* < 0.05), but not on ΔEF (*p* > 0.05; Supplementary Figure S3).

### Relationship between WMH, CSF Aβ42 levels, and traditional neurodegeneration markers

**Figure 7:**
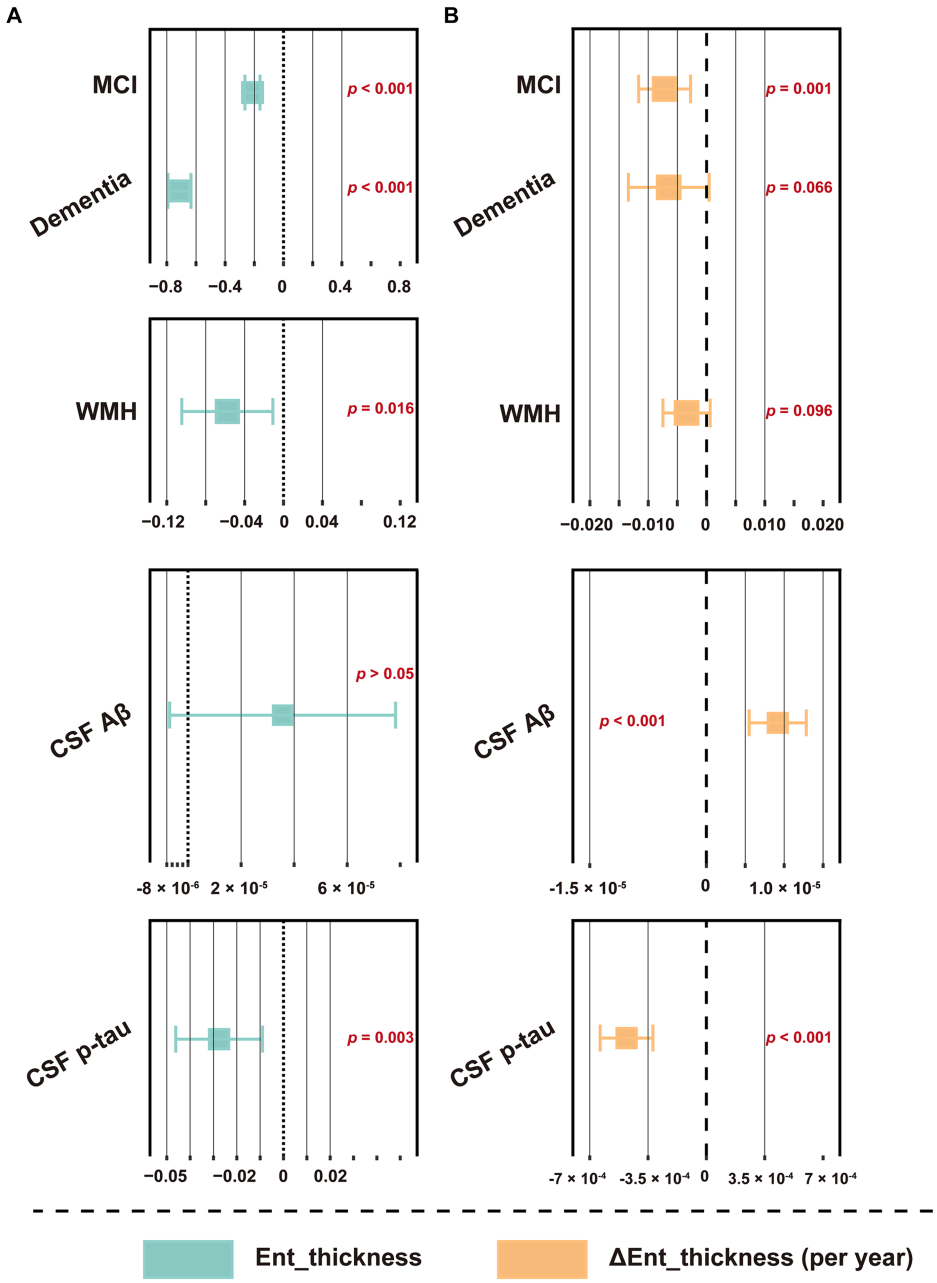
Forest plot of the association between WMH volume and entorhinal cortex thickness. (**A**) The association between baseline WMH volume and entorhinal cortex thickness. (**B**) The association between baseline WMH volume and the changes slope of entorhinal cortex thickness. Baseline WMH volume, plus diagnostic status, age, sex, education, *APOE* ε4 status, hypertension and smoking status, and CSF core biomarkers, including Aβ_42_ and p-tau levels were used as predictors of cognitive function slopes. The WMH volume was total intracranial volume-normalized and log-transformed. CSF t-tau was not included due to its extremely high correlation with p-tau (*R* > 0.900, *p* < 0.001). For effect estimates with exact 95% CI and statistical significance values, see Supplementary Table S13. MCI, mild cognitive impairment; WMH, white matter hyperintensity; CSF, cerebrospinal fluid; Aβ, β-amyloid; p-tau, phosphorylated tau; t-tau, total tau; APOE, apolipoprotein E; MEM, memory sub-domain; EF, executive function; ADNI, Alzheimer’s Disease Neuroimaging Initiative; CI, confidence interval.

CSF GAP43 and t-tau, FDG SUVR, and structural indicators, including hippocampal volume, cerebral gray matter volume, and entorhinal cortex thickness, are commonly considered as markers of neurodegeneration, reflecting nerve damage from different perspectives (Jack et al., 2018; Zhang et al., 2022). Herein, we explored their relationship with WMH. At the cross-sectional level, the results indicated that baseline WMH volume had a weak but significant correlation with these markers, except for CSF GAP43 (Supplementary Figure S4; |R| = 0.078–0.295; all *p* < 0.05). Longitudinal analysis further showed that WMH was only correlated with the slope of hippocampal volume and entorhinal cortex thickness (Supplementary Figure S4; *R* = −0.067 and − 0.228, respectively; *p* = 0.030 and *p* < 0.001, respectively). The correlation between CSF AD core features and these indicators was generally more prominent. Further linear regression was corrected for the influence of confounding factors, and the results indicated that WMH could still independently related to the thickness of the entorhinal cortex (baseline level, *p* = 0.016; change slope, approaching significance, *p* = 0.096; see Figure 7 and Supplementary Table S13 for details), but not other indicators (data not shown). Similar results were obtained in individuals without dementia (Supplementary Figure S4).

## Discussion

In this study, we used data from a large cohort of participants along the cognitive continuum, to investigate WMH in detail. Overall, our analysis revealed the following results: (i) age and CSF Aβ_42_ levels were the most important factors related to WMH volume, and these results remained largely consistent across all clinical stages, although hypertension and smoking history were only associated with WMH in non-dementia participants; (ii) higher WMH volume at baseline was associated with worse cognitive performance and a more rapid memory decline, however, baseline WMH volume cannot predict future dementia conversion; (iii) participants with dementia, or non-dementia participants harboring cerebral Aβ both exhibited a greater accrual of WMH volume, and a greater increase in WMH volume was found to be linked with a more rapid cognitive decline; (iv) to a lesser extent, the correlation between cerebral Aβ and cognition was affected by the mediating effects of WMH; (v) among different neurodegeneration markers, the WMH was only independently and significantly associated with the thickness of entorhinal cortex.

WMH are generally considered to be of vascular origin, and have been related to blood–brain barrier abnormalities and neuroinflammation (Garnier-Crussard et al., 2023; Ying et al., 2023). Previous studies have confirmed the correlation between WMH and age (Yoshita et al., 2006; Wei et al., 2019; Walsh et al., 2020; Luo et al., 2023; Ortner et al., 2023), as well as overall vascular risk factors (Yoshita et al., 2006; Cox et al., 2019; Alban et al., 2023). In several significantly large cohorts, researchers have indicated that arterial hypertension is associated with a larger WMH volume and accelerated WMH progression (Goldstein et al., 2005; Gottesman et al., 2010; Verhaaren et al., 2013; Bernal et al., 2023). The association between smoking and elevated WMH has also been confirmed in a large study of the UK Biobank samples (Gray et al., 2020). However, it should be noted that these studies mainly focused on participants free of dementia. This study did not identify any link between WMH and hypertension or smoking in the dementia group, emphasizing the importance of early intervention. Smoking and blood pressure may exert their effects through endothelial damage, oxidative stress and inflammatory mechanisms, and may have potential for dementia prevention due to their modifiable nature (Silva et al., 2022). Although diabetes, atrial fibrillation, and hyperlipidemia are all potential risk factors for atherosclerosis and brain infarction, the present study did not identify any relationship with WMH. In fact, several prior studies have shown no or only a weak association between diabetes and WMH (Prins and Scheltens, 2015; Moroni et al., 2020). Regarding hyperlipidemia, both positive and negative correlations have been observed with WMH (Moroni et al., 2020). Stroke may promote the occurrence of WMH (Loos et al., 2018; Duering et al., 2023), but this study did not find any association between cardiovascular and cerebrovascular diseases and WMH. From our perspective, these inconsistent results may be related to the study design and cohort differences. For example, people with stroke and other cardiovascular diseases were generally excluded from ADNI and thus it has less cardio- and cerebrovascular disease burden than the general older adults. Further research is still needed.

Cortical amyloid deposition is another key contributor to the development of WMH. Despite contradictions by some studies (Hedden et al., 2012; Marchant et al., 2013; Park et al., 2014; Luo et al., 2023; Ortner et al., 2023; Ying et al., 2023), we believe that our results were reliable because the correlations between CSF Aβ_42_ levels and WMH volume were not affected by confounding factors, and were significant at all clinical stages, suggesting that WMH could also be AD origin and another core feature of AD (Iturria-Medina et al., 2016; Lee et al., 2016). Recent studies have further found that the cerebral Aβ burden was associated with WMH located in specific posterior regions, indicating a region-specific effect (Weaver et al., 2019; Bernal et al., 2023). Furthermore, a potentially self-reinforcing mechanism has been proposed, indicating that vascular pathologies may initiate Aβ deposition on the one hand, while on the other hand, Aβ deposition may also increase the susceptibility to developing vascular lesions (Klohs, 2019). Specifically, vascular dysfunction could promote oxidative stress and neuroinflammation, thereby stimulating the increased production and limiting the clearance of Aβ (Rost et al., 2022). To some extent, this effect can be achieved through platelet activation (Li and Liu, 2022). Conversely, due to the toxic effects on pericytes and endothelial cells, elevations in Aβ have been shown to cause vasoconstriction, reduce cerebral perfusion, and prevent functional hyperemia and autoregulation, finally resulting in lesions including WMH (Love and Miners, 2016; Cortes-Canteli and Iadecola, 2020).

There is significant evidence to show that cerebrovascular disease accelerates the onset of dementia in AD patients (Snowdon et al., 1997; Cortes-Canteli and Iadecola, 2020; Rost et al., 2022). Individuals with mixed findings of vascular and AD pathologies are almost three times more likely to develop dementia than those with a single pathological diagnosis (Schneider et al., 2007). This study found that higher WMH volume at baseline was associated with worse cognitive performance and a faster memory decline. However, the Cox regression results suggested that WMH volume cannot predict future dementia conversion, which was inconsistent with previous studies (Debette and Markus, 2010; Gordon et al., 2015; Hu et al., 2021). Our negative findings may be related to the short follow-up time. Furthermore, we made longitudinal analyses of WMH volume, which showed that baseline features of AD (including dementia diagnosis and positive Aβ status) were associated with future increase in WMH volume, and that longitudinal WMH volume were correlated with further change in cognition. Specifically, faster WMH volume aggregation reflects a more rapid deterioration of future cognition. These results suggest that longitudinal WMH volume can be used to dynamically track neurodegeneration throughout the preclinical stage and different clinical stages of AD. Co-pathologies lead to uncertainty regarding the role of WMH. As such, we corrected for confounding factors including CSF Aβ and p-tau. After these analyses, the relationships between WMH/ΔWMH and cognition was still significant, indicating that the long-term effects occurred independent of AD pathologies. In contrast, previous studies either did not consider confounding factors, or corrected only for demographic information (Debette and Markus, 2010; Hu et al., 2021). Our recent review suggested that Aβ could damage cognitive function independent of tau pathology and neurodegeneration; however, the specific mechanism remains unclear (Li et al., 2022b). This study found that WMH mediates the correlation between brain Aβ deposition and cognition. It should be noted that although these mediating effects were statistically significant, the degree was very weak, indicating that Aβ acts primarily through other mechanisms (Jack et al., 2018). Recent imaging studies have indicated that WMH may cause cognitive impairment by interrupting the connection between the cortex and subcortical nucleus (Zhu et al., 2023). In addition, WMH-associated microglial activation plays a role in the development of AD (Prins and Scheltens, 2015; Moroni et al., 2020).

How WMH relate to different markers of neurodegeneration is currently only poorly understood, as previous studies either did not pay much attention to the potential interactions of AD core pathologies, or focused on only a single marker instead of investigating multiple neurodegenerative markers using various modalities. Herein, we analyzed markers from the CSF, as well as those extracted from PET and structural MRI. Overall, our results showed that the correlations between markers and WMH were affected by Aβ or tau pathologies to a certain extent, and WMH was only independently associated with the thickness of entorhinal cortex. Previously, Bos et al. found that WMH and Aβ are independent determinants of medial temporal lobe atrophy and that their effects are additive (Bos et al., 2017). However, the authors regarded both WMH and Aβ as binary variables, rather than continuous variables, and estimated atrophy subjectively rather than using objective data, which may have resulted in a poor robustness. Similar to our results, Kurz et al. found that elevated t-tau levels in patients with vascular damage could occur as a result of coexisting Aβ pathology (Kurz et al., 2003). In one small sample, researchers also found no correlation between WMH volume and the FDG SUVR (Ortner et al., 2023). The correlation mechanism between WMH and cortical thickness needs further exploration.

This study had several limitations. First, we defined previous chronic diseases and smoking history based on a retrospective screening of medical records, which could have missed newly diagnosed patients, and we did not consider the severity and control of disease (or smoking). Secondly, considering its rarity, we did not screen for mutations related to CSVD. Third, this study focused on the total volume of WMH, rather than its pattern (punctuated or confluent) or location (deep or periventricular), although some prior studies have shown that WMH from different sources may have distinct patterns (Keller et al., 2023) or locations (Weaver et al., 2019). Fourth, based on the ADNI’s inclusion/exclusion criteria, participants were more likely to identify their race as White, highly educated, and *APOE* ε4 positive, and had relatively low vascular risk burdens (Gianattasio et al., 2021). The impact of race on the burden of AD pathologies may be severely underestimated (Royse et al., 2021). Thus, findings from our study may not be assumed to be directly generalizable to other populations. Future research should therefore include participants with greater racial diversity and educational attainments. Fifth, loss to follow-up remains a concern in longitudinal study, which may have introduced selection bias. Sixth, longitudinal mediation studies are warranted to underpin the relations among variables over time and establish causality. Seventh, WMH’s mechanism of action of WMHs was not investigated. Finally, compared with subjective ratings (Gordon et al., 2015; Marnane et al., 2016; van Westen et al., 2016), this study used an automated quantitative method to calculate WMH volume; as such, it should be noted that different evaluation methods may lead to different results.

## Conclusion

WMH are non-specific lesions associated with both vascular and AD factors. Increased baseline volume and accruals were both found to be associated with cognitive decline. Importantly, we found that their impacts on cognition were independent of Aβ, and these two pathologies are not simply additive. WMH mediates the correlation between Aβ and cognition, and there is likely to be a mutually reinforcing relationship (Klohs, 2019; Cortes-Canteli and Iadecola, 2020; Rost et al., 2022). For neurodegeneration, we confirmed that WMH volume was only associated with the thickness of entorhinal cortex, not other markers. Owing to the particular impact of WMH on cognition, the present study highlights the value of incorporating vascular pathologies into AD biomarkers. Both vascular and AD pathologies should be considered when developing therapeutic targets. Given the limited treatment options for dementia, early intervention to reduce vascular lesions is a high priority.

## Data Availability

Publicly available datasets were analyzed in this study. This data can be found here: http://adni.loni.usc.edu/. Please note that access is contingent on adherence to the ADNI Data Use Agreement and the publications’ policies.

## Footnotes

^1^http://surfer.nmr.mgh.harvard.edu/

^2^http://adni.loni.usc.edu

## The Alzheimer’s disease neuroimaging initiative

Data used in the preparation of this article were obtained from the Alzheimer’s Disease Neuroimaging Initiative (ADNI) database (http://adni.loni.usc.edu). As such, the investigators within the ADNI contributed to the design and implementation of ADNI and/or provided data but did not participate in analysis or writing of this report. A complete listing of ADNI investigators can be found at: http://adni.loni.usc.edu/wp-content/uploads/how_to_apply/ADNI_Acknowledgement_List.pdf.
