# Supplementary material for: Association of white matter hyperintensities with cognitive decline and neurodegeneration
Source: Front Aging Neurosci. 2024 Sep 12;16:1412735. doi: 10.3389/fnagi.2024.1412735 (PMC11425965; doi:10.3389/fnagi.2024.1412735)
Supplement: Supplementary file 1 [file Data_Sheet_1.PDF]

Supplementary Information for

Effects of White Matter Hyperintensities on Cognitive Decline and Neurodegeneration

Table of Contents

**Content**	**Page**
**Supplementary Tables**	
**Table S1**. Baseline participant characteristics *(n = 1803; total)*	**2**
**Table S2**. Baseline participant characteristics *(additionally by "A" status)*	**4**
**Table S3**. Baseline participant characteristics *(additionally by "T" status)*	**6**
**Table S4**. Baseline participant characteristics *(additionally by "A/T" status)*	**8**
**Table S5**. Baseline participant characteristics *(n = 1056; non-dementia)*	**11**
**Table S6**. Relationships between brain WMH volume and AD CSF core biomarkers	**13**
**Table S7**. Cox regression analysis for dementia conversion from 876 non-dementia participants	**14**
**Table S8.** The proportional hazards assumption was tested using the scaled *Schoenfeld* residuals	**15**
**Table S9**. Relationships between baseline brain WMH volume and cognitive function slopes	**16**
**Table S10**. Change in WMH volume within the next 48 months in different cognitive groups	**17**
**Table S11**. Change in WMH volume within the next 48 months in the groups with different Aβ status	**17**
**Table S12**. Relationships between ∆WMH volume and cognitive function slopes	**18**
**Table S13**. Relationships between baseline brain WMH volume and entorhinal cortex thickness	**18**
**Supplementary Figures**	
**Figure S1**. Correlation analysis matrices	**19**
**Figure S2**. WMH volume in different diagnostic groups	**20**
**Figure S3**. Mediation analyses of brain Aβ deposition on cognitive function slopes	**21**
**Figure S4**. Correlation analysis matrices *(WMH volume and neurodegeneration markers)*	**22**

Supplementary Table 1. Baseline participant characteristics

	NC (N = 756)		MCI (N = 783)		Dementia (N = 264)	
	Mean ± SD (or %)	n	Mean ± SD (or %)	n	Mean ± SD (or %)	n
**age at baseline**	72.0 ± 7.25	756	72.4 ± 7.85$^{\#}$	780	75.4 ± 8.21***	263
**male**	312 (41.3%)	756	430 (55.1%)***	780	151 (57.4%)***	263
**education**	16.6 ± 2.42	756	16.2 ± 2.60**	780	15.6 ± 2.74***	263
***APOE* ε4-/-**	434 (68.8%)	631	348 (53.8%)***	647	73 (32.6%)***	224
***APOE* ε4+/-**	181 (28.7%)	631	234 (36.2%)**	647	106 (47.3%)***	224
***APOE* ε4+/+**	16 (2.5%)	631	65 (10.0%)***	647	45 (20.1%)***	224
**Past medical histories**						
**Hypertension**	342 (45.5%)	751	393 (50.2%)$^{\#}$	783	131 (49.8%)$^{\#}$	263
**Diabetes**	103 (13.7%)	751	99 (12.6%)$^{\#}$	783	44 (16.7%)$^{\#}$	263
**Hyperlipidemia**	387 (51.5%)	751	406 (51.9%)$^{\#}$	783	152 (57.8%)$^{\#}$	263
**Smoking**	181 (24.1%)	751	244 (31.2%)**	783	85 (32.3%)**	263
**Atrial fibrillation**	32 (4.3%)	751	39 (5.0%)$^{\#}$	783	10 (3.8%)$^{\#}$	263
**Coronary artery diseases**	70 (9.3%)	751	91 (11.6%)$^{\#}$	783	31 (11.8%)$^{\#}$	263
**Cerebrovascular diseases**	25 (3.3%)	751	36 (4.6%)$^{\#}$	783	7 (2.7%)$^{\#}$	263
**Neuropsychological scales**						
**MMSE**	29.05 ± 1.172	755	27.89 ± 1.873***	783	22.74 ± 3.013***	264
**MoCA**	25.71 ± 2.657	696	23.02 ± 3.297***	709	16.57 ± 4.672***	239
**ADAS-cog13**	8.76 ± 4.371	697	15.02 ± 6.707***	708	31.48 ± 8.801***	235
**Logical Memory**	13.19 ± 3.375	701	7.16 ± 3.456***	711	1.59 ± 2.219***	239
**ADNI_MEM**	1.04 ± 0.604	704	0.34 ± 0.667***	714	-0.91 ± 0.587***	241
**ADNI_EF**	0.95 ± 0.828	697	0.35 ± 0.895***	711	-0.91 ± 1.015***	237
**ADNI_LAN**	0.84 ± 0.721	704	0.34 ± 0.775***	714	-0.79 ± 0.966***	241
**ADNI_VS**	0.18 ± 0.645	704	-0.03 ± 0.754***	714	-0.63 ± 1.058***	241
**CSF core biomarkers**						
**Aβ_{42} (pg/mL)**	1364.57 ± 646.786	474	1088.86 ± 584.034***	542	697.20 ± 408.758***	166
**p-tau (pg/mL)**	21.47 ± 9.496	474	26.38 ± 14.684***	542	36.62 ± 15.670***	166
**t-tau (pg/mL)**	236.54 ± 90.254	474	274.53 ± 129.033***	542	370.43 ± 147.776***	166
**CSF GAP43 (pg/mL)**	4996.59 ± 2690.805	248	5122.34 ± 2835.664$^{\#}$	412	6312.11 ± 3131.703***	126
**Neuroimaging**						
**FDG (metaROI SUVR)**	1.30 ± 0.054	314	1.26 ± 0.075***	653	1.14 ± 0.114***	214
**B_HP volume (cm^3)**	6.64 ± 0.768	756	6.35 ± 0.888***	783	5.46 ± 0.914***	264
**GM volume (cm^3)**	510.05 ± 49.250	756	503.26 ± 50.263*	783	480.28 ± 52.747***	264
**Ent_thickness (mm)**	3.58 ± 0.302	756	3.36 ± 0.459***	783	2.77 ± 0.517***	264
**WMH volume (cm^3)**	4.77 ± 8.637	756	6.48 ± 8.894**	783	8.71 ± 12.397***	264
**WMH volume (Logarithmization)^{a}**	0.28 ± 0.627	756	0.48 ± 0.595***	783	0.66 ± 0.528***	264
**WMH volume (Logarithmization)^{b}**	-2.87 ± 0.626	756	-2.67 ± 0.592***	783	-2.49 ± 0.522***	264
**Participants with follow-up WMH data within 48 months**						
**N at baseline**	501		587		175	
**N at 12 months**	285		527		136	
**N at 24 months**	367		415		48	
**N at 36 months**	43		139		3	
**N at 48 months**	160		198		0	

The included 1803 participants were grouped according to their clinical diagnoses. Categorical and continuous measures are presented as numbers (%) or means ± standard deviations. The statistical analyses were conducted by chi-square tests for categorical variables and one-way ANOVAs followed by Tukey's test for continuous variables (adjusted *p* value). Compared with the NC group: *, < 0.05; **, < 0.01; ***, < 0.001; #, > 0.05. **Coronary artery diseases** include patients who have (or have not) undergone stent placement or bypass grafting. **Cerebrovascular diseases** refer to transient ischemic attack and ischemic stroke. **Logical memory** indicates the total number of story units that are recalled. **ADNI_MEM/EF/LAN/VS** refers to the composite measures of memory function/executive function/language function/visuospatial function derived from the ADNI database. **MetaROI** includes the left angular gyrus, right angular gyrus, bilateral posterior cingulate gyrus, left inferior temporal gyrus, and right inferior temporal gyrus, which are the most important hypometabolic regions indicative of pathological metabolic changes in patients with AD. **B_HP volume** indicates the total volume of bilateral hippocampus. **GM volume** indicates the cerebral gray matter volume. **Ent_thickness** indicates the average thickness of bilateral entorhinal cortices. **WMH volume (Logarithmization)** means natural log-transformed WMH (a) or log-transformed total intracranial volume-normalized WMH (b).

Abbreviations: NC, cognitively normal control; MCI, mild cognitive impairment; APOE, apolipoprotein E; MMSE, Mini-Mental State Examination; MoCA, Montreal Cognitive Assessment; ADAS-Cog 13, Alzheimer's Disease Assessment Scale-Cognitive 13; ADNI, Alzheimer's Disease Neuroimaging Initiative; MEM, memory sub-domain; EF, executive function sub-domain; LAN, language sub-domain; VSP, visuospatial sub-domain; CSF, cerebrospinal fluid; Aβ, β-amyloid; p-tau, phosphorylated tau; t-tau, total tau; GAP43, growth-associated protein-43; FDG, [^{18}F]fluoro-2-deoxyglucose; ROI, region of interest; SUVR, standardized uptake value ratio; B, bilateral; HP, hippocampus; WMH, white matter hyperintensity; GM, gray matter; Ent, entorhinal cortex; ANOVA, analysis of variance; AD, Alzheimer's disease.

Supplementary Table 2. Baseline participant characteristics

	A- NC (N = 317)		A+ NC (N = 157)		A- MCI (N = 247)		A+ MCI (N = 295)		A- dementia (N = 17)		A+ dementia (N = 149)	
**n**	**Mean ± SD (or %)**	**n**	**Mean ± SD (or %)**	**n**	**Mean ± SD (or %)**	**n**	**Mean ± SD (or %)**	**n**	**Mean ± SD (or %)**	**n**	**Mean ± SD (or %)**	**n**
**age at baseline**	71.9 ± 6.53	317	73.1 ± 6.52	157	70.2 ± 7.74	247	72.9 ± 7.08	295	77.6 ± 7.54	17	73.7 ± 8.44	149
**male**	131 (41.3%)	317	66 (42.0%)	157	125 (50.6%)	247	178 (60.3%)	295	14 (82.4%)	17	87 (58.4%)	149
**education**	16.9 ± 2.38	317	16.6 ± 2.25	157	16.2 ± 2.50	247	16.2 ± 2.70	295	16.5 ± 2.18	17	15.7 ± 2.66	149
***APOE* ε4-/-**	245 (77.5%)	316	74 (48.4%)	153	183 (76.6%)	239	96 (33.4%)	287	11 (64.7%)	17	41 (27.7%)	148
***APOE* ε4+/-**	69 (21.8%)	316	66 (43.1%)	153	53 (22.2%)	239	135 (47.0%)	287	5 (29.4%)	17	75 (50.7%)	148
***APOE* ε4+/+**	2 (0.6%)	316	13 (8.5%)	153	3 (1.3%)	239	56 (19.5%)	287	1 (5.9%)	17	32 (21.6%)	148
**Past medical histories**												
**Hypertension**	134 (42.4%)	316	76 (48.7%)	156	125 (50.6%)	247	143 (48.5%)	295	11 (64.7%)	17	67 (45.3%)	148
**Diabetes**	41 (13.0%)	316	13 (8.3%)	156	33 (13.4%)	247	29 (9.8%)	295	5 (29.4%)	17	18 (12.2%)	148
**Hyperlipidemia**	166 (52.5%)	316	83 (53.2%)	156	130 (52.6%)	247	159 (53.9%)	295	8 (47.1%)	17	82 (55.4%)	148
**Smoking**	86 (27.2%)	316	42 (26.9%)	156	74 (30.0%)	247	107 (36.3%)	295	8 (47.1%)	17	49 (33.1%)	148
**Atrial fibrillation**	12 (3.8%)	316	7 (4.5%)	156	5 (2.0%)	247	12 (4.1%)	295	2 (11.8%)	17	3 (2.0%)	148
**CADs**	23 (7.3%)	316	20 (12.8%)	156	26 (10.5%)	247	35 (11.9%)	295	3 (17.6%)	17	17 (11.5%)	148
**CDs**	13 (4.1%)	316	5 (3.2%)	156	9 (3.6%)	247	13 (4.4%)	295	1 (5.9%)	17	4 (2.7%)	148
**Neuropsychological scales**												
**MMSE**	29.15 ±1.117	317	29.01 ± 1.182	157	28.41 ± 1.587	247	27.69 ± 1.946	295	24.00 ± 1.658	17	22.87 ± 2.332	149
**MoCA**	26.20 ± 2.501	315	25.35 ± 2.637	156	24.05 ± 2.956	245	22.41 ± 3.149	294	19.06 ± 4.160	17	16.62 ± 4.727	148
**ADAS-cog13**	8.32 ± 4.262	314	8.97 ± 4.617	157	12.45 ± 5.667	245	16.99 ± 6.665	294	25.88 ± 5.500	17	31.81 ± 8.535	145
**Logical Memory**	13.70 ± 3.317	317	12.96 ± 2.949	157	8.12 ± 2.780	245	6.18 ± 3.456	295	2.12 ± 2.736	17	1.55 ± 1.805	148
**ADNI_MEM**	1.11 ± 0.571	317	0.97 ± 0.601	157	0.62 ± 0.623	247	0.10 ± 0.588	295	-0.60 ± 0.398	17	-0.90 ± 0.521	149
**ADNI_EF**	1.11 ± 0.820	317	0.79 ± 0.807	157	0.67 ± 0.811	247	0.15 ± 0.857	294	-0.32 ± 0.958	17	-0.92 ± 1.023	146
**ADNI_LAN**	0.96 ± 0.719	317	0.83 ± 0.707	157	0.53 ± 0.683	247	0.19 ± 0.778	295	-0.41 ± 0.753	17	-0.75 ± 0.931	149
**ADNI_VS**	0.22 ± 0.626	317	0.15 ± 0.649	157	0.08 ± 0.691	247	-0.10 ± 0.761	295	-0.61 ± 0.975	17	-0.63 ± 1.065	149
**CSF core biomarkers**												
**Aβ_{42} (pg/mL)**	1684.99 ± 547.706	317	717.61 ± 175.707	157	1598.88 ± 484.778	247	661.82 ± 171.770	295	1624.95 ± 666.562	17	591.35 ± 167.619	149
**p-tau (pg/mL)**	20.69 ± 7.828	317	23.04 ± 12.066	157	21.16 ± 10.384	247	30.75 ± 16.261	295	29.62 ± 17.456	17	37.42 ± 15.315	149
**t-tau (pg/mL)**	235.55 ± 82.259	317	238.52 ± 104.814	157	236.34 ± 96.979	247	306.51 ± 143.235	295	339.02 ± 177.076	17	374.01 ± 144.327	149

**CSF GAP43 (pg/mL)**	5161.84 ± 2569.063	166	4662.04 ± 2909.462	82	4628.12 ± 2390.232	187	5533.10 ± 3104.254	225	7226.16 ± 3038.797	14	6197.85 ± 3137.597	112
**Neuroimaging**												
**FDG (metaROI SUVR)**	1.30 ± 0.054	177	1.29 ± 0.054	88	1.29 ± 0.065	240	1.25 ± 0.078	288	1.23 ± 0.077	17	1.13 ± 0.116	144
**B_HP volume (cm^3)**	6.72 ± 0.773	317	6.65 ± 0.687	157	6.51 ± 0.921	247	6.24 ± 0.872	295	5.00 ± 0.901	17	5.55 ± 0.883	149
**GM volume (cm^3)**	510.84 ± 50.713	317	503.8 ± 46.374	157	501.39 ± 47.828	247	497.31 ± 50.882	295	483.39 ± 43.601	17	480.65 ± 56.84	149
**Ent_thickness (mm)**	3.59 ± 0.294	317	3.57 ± 0.284	157	3.44 ± 0.468	247	3.28 ± 0.451	295	2.76 ± 0.621	17	2.78 ± 0.485	149
**WMH volume (cm^3)**	3.67 ± 4.819	317	6.90 ± 12.802	157	5.06 ± 7.326	247	7.44 ± 9.215	295	4.76 ± 6.318	17	8.30 ± 9.897	149
**WMH volume (Logarithmization)[a]**	0.25 ± 0.579	317	0.47 ± 0.583	157	0.37 ± 0.575	247	0.60 ± 0.531	295	0.46 ± 0.416	17	0.67 ± 0.509	149
**WMH volume (Logarithmization)[b]**	-2.90 ± 0.578	317	-2.68 ± 0.580	157	-2.78 ± 0.575	247	-2.55 ± 0.527	295	-2.68 ± 0.420	17	-2.48 ± 0.498	149

The analysis was performed in a subset of the included 1803 participants, these individuals had available CSF data and were grouped according to their clinical diagnoses and amyloid status (n = 1182). According to a previous standard, we set the cutoff value at 977 pg/mL for Aβ to select participants with Aβ deposition (< 977 pg/mL; A+). Categorical and continuous measures are presented as numbers (%) or means ± standard deviations. **CADs** include patients who have (or have not) undergone stent placement or bypass grafting. **CDs** refer to transient ischemic attack and ischemic stroke. **Logical memory** indicates the total number of story units that are recalled. **ADNI_MEM/EF/LAN/VS** refers to the composite measures of memory function/executive function/language function/visuospatial function derived from the ADNI database. **MetaROI** includes the left angular gyrus, right angular gyrus, bilateral posterior cingulate gyrus, left inferior temporal gyrus, and right inferior temporal gyrus, which are the most important hypometabolic regions indicative of pathological metabolic changes in patients with AD. **B_HP volume** indicates the total volume of bilateral hippocampus. **GM volume** indicates the cerebral gray matter volume. **Ent_thickness** indicates the average thickness of bilateral entorhinal cortices. **WMH volume (Logarithmization)** means natural log-transformed WMH (a) or log-transformed total intracranial volume-normalized WMH (b).

Abbreviations: NC, cognitively normal control; MCI, mild cognitive impairment; APOE, apolipoprotein E; CADs, Coronary artery diseases; CDs, Cerebrovascular diseases; MMSE, Mini-Mental State Examination; MoCA, Montreal Cognitive Assessment; ADAS-Cog 13, Alzheimer's Disease Assessment Scale-Cognitive 13; ADNI, Alzheimer's Disease Neuroimaging Initiative; MEM, memory sub-domain; EF, executive function sub-domain; LAN, language sub-domain; VSP, visuospatial sub-domain; CSF, cerebrospinal fluid; Aβ, β-amyloid; p-tau, phosphorylated tau; t-tau, total tau; GAP43, growth associated protein-43; FDG, [^{18}F]fluoro-2-deoxyglucose; ROI, region of interest; SUVR, standardized uptake value ratio; B, bilateral; HP, hippocampus; WMH, white matter hyperintensity; GM, gray matter; Ent, entorhinal cortex; AD, Alzheimer's disease.

Supplementary Table 3. Baseline participant characteristics

	T- NC (N = 372)		T+ NC (N = 102)		T- MCI (N = 348)		T+ MCI (N = 194)		T- dementia (N = 50)		T+ dementia (N = 116)	
**n**	**Mean ± SD (or %)**	**n**	**Mean ± SD (or %)**	**n**	**Mean ± SD (or %)**	**n**	**Mean ± SD (or %)**	**n**	**Mean ± SD (or %)**	**n**	**Mean ± SD (or %)**	**n**
**age at baseline**	71.4 ± 6.22	372	75.6 ± 6.66	102	70.5 ± 7.56	348	73.7 ± 6.99	194	74.7 ± 8.21	50	73.8 ± 8.53	116
**male**	156 (41.9%)	372	41 (40.2%)	102	199 (57.2%)	348	104 (53.6%)	194	38 (76.0%)	50	63 (54.3%)	116
**education**	16.8 ± 2.32	372	16.6 ± 2.42	102	16.3 ± 2.63	348	16.1 ± 2.56	194	16.7 ± 2.72	50	15.4 ± 2.50	116
***APOE* ε4-/-**	265 (72.2%)	367	54 (52.9%)	102	215 (64.0%)	336	64 (33.7%)	190	19 (38.0%)	50	33 (28.7%)	115
***APOE* ε4+/-**	92 (25.1%)	367	43 (42.2%)	102	94 (28.0%)	336	94 (49.5%)	190	23 (46.0%)	50	57 (49.6%)	115
***APOE* ε4+/+**	10 (2.7%)	367	5 (4.9%)	102	27 (8.0%)	336	32 (16.8%)	190	8 (16.0%)	50	25 (21.7%)	115
**Past medical histories**												
**Hypertension**	165 (44.5%)	371	45 (44.6%)	101	180 (51.7%)	348	88 (45.4%)	194	28 (56.0%)	50	50 (43.5%)	115
**Diabetes**	41 (11.1%)	371	13 (12.9%)	101	42 (12.1%)	348	20 (10.3%)	194	10 (20.0%)	50	13 (11.3%)	115
**Hyperlipidemia**	183 (49.3%)	371	66 (65.3%)	101	183 (52.6%)	348	106 (54.6%)	194	28 (56.0%)	50	62 (53.9%)	115
**Smoking**	99 (26.7%)	371	29 (28.7%)	101	120 (34.5%)	348	61 (31.4%)	194	17 (34.0%)	50	40 (34.8%)	115
**Atrial fibrillation**	15 (4.0%)	371	4 (4.0%)	101	12 (3.4%)	348	5 (2.6%)	194	4 (8.0%)	50	1 (0.9%)	115
**CADs**	34 (9.2%)	371	9 (8.9%)	101	34 (9.8%)	348	27 (13.9%)	194	8 (16.0%)	50	12 (10.4%)	115
**CDs**	15 (4.0%)	371	3 (3.0%)	101	15 (4.3%)	348	7 (3.6%)	194	1 (2.0%)	50	4 (3.5%)	115
**Neuropsychological scales**												
**MMSE**	29.11 ± 1.110	372	29.07 ± 1.245	102	28.35 ± 1.637	348	27.42 ± 1.991	194	23.46 ± 2.062	50	22.78 ± 2.366	116
**MoCA**	26.04 ± 2.509	370	25.47 ± 2.770	101	23.77 ± 2.959	347	22.04 ± 3.232	192	17.86 ± 4.580	50	16.44 ± 4.734	115
**ADAS-cog13**	8.44 ± 4.471	369	8.88 ± 4.082	102	13.15 ± 5.664	346	18.11 ± 7.028	193	28.01 ± 7.241	48	32.52 ± 8.598	114
**Logical Memory**	13.44 ± 3.269	372	13.52 ± 3.030	102	7.87 ± 2.886	348	5.59 ± 3.523	192	1.70 ± 2.178	50	1.57 ± 1.802	115
**ADNI_MEM**	1.09 ± 0.592	372	0.97 ± 0.547	102	0.52 ± 0.606	348	0.01 ± 0.615	194	-0.75 ± 0.494	50	-0.93 ± 0.520	116
**ADNI_EF**	1.03 ± 0.809	372	0.90 ± 0.893	102	0.55 ± 0.847	347	0.10 ± 0.852	194	-0.75 ± 1.161	49	-0.91 ± 0.970	114
**ADNI_LAN**	0.94 ± 0.706	372	0.83 ± 0.755	102	0.48 ± 0.749	348	0.10 ± 0.700	194	-0.50 ± 0.844	50	-0.82 ± 0.936	116
**ADNI_VS**	0.21 ± 0.632	372	0.14 ± 0.644	102	0.06 ± 0.713	348	-0.16 ± 0.754	194	-0.59 ± 0.958	50	-0.64 ± 1.096	116
**CSF core biomarkers**												
**$A\beta_{42}$ (pg/mL)**	1379.98 ± 592.334	372	1308.38 ± 816.244	102	1203.20 ± 582.923	348	883.76 ± 528.525	194	732.72 ± 451.658	50	681.90 ± 389.903	116
**p-tau (pg/mL)**	17.50 ± 4.682	372	35.95 ± 8.476	102	17.98 ± 4.885	348	41.45 ± 14.345	194	21.29 ± 4.468	50	43.23 ± 14.055	116
**t-tau (pg/mL)**	199.22 ± 50.561	372	372.64 ± 70.158	102	200.93 ± 51.015	348	406.55 ± 121.258	194	231.60 ± 47.874	50	430.27 ± 135.514	116

**CSF GAP43 (pg/mL)**	4215.58 ± 1943.137	192	7674.34 ± 3158.130	56	3802.49 ± 1550.586	260	7380.00 ± 3103.573	152	4322.94 ± 2083.999	41	7271.59 ± 3108.637	85
**Neuroimaging**												
**FDG (metaROI SUVR)**	1.30 ± 0.052	204	1.29 ± 0.059	61	1.28 ± 0.070	338	1.24 ± 0.077	190	1.16 ± 0.128	48	1.13 ± 0.111	113
**B_HP volume (cm^3)**	6.73 ± 0.762	372	6.58 ± 0.674	102	6.52 ± 0.881	348	6.09 ± 0.882	194	5.72 ± 0.868	50	5.39 ± 0.896	116
**GM volume (cm^3)**	508.09 ± 50.000	372	510.04 ± 47.261	102	502.98 ± 48.558	348	492.34 ± 50.586	194	501.58 ± 47.633	50	472.03 ± 56.492	116
**Ent_thickness (mm)**	3.60 ± 0.287	372	3.55 ± 0.303	102	3.44 ± 0.442	348	3.21 ± 0.471	194	2.93 ± 0.530	50	2.71 ± 0.471	116
**WMH volume (cm^3)**	4.18 ± 5.770	372	6.79 ± 14.455	102	5.87 ± 8.289	348	7.24 ± 8.775	194	9.29 ± 11.255	50	7.36 ± 8.842	116
**WMH volume (Logarithmization)**[a]	0.29 ± 0.586	372	0.42 ± 0.587	102	0.43 ± 0.576	348	0.61 ± 0.522	194	0.72 ± 0.498	50	0.62 ± 0.505	116
**WMH volume (Logarithmization)**[b]	-2.85 ± 0.586	372	-2.73 ± 0.585	102	-2.72 ± 0.572	348	-2.54 ± 0.521	194	-2.45 ± 0.494	50	-2.53 ± 0.494	116

The analysis was performed in a subset of the included 1803 participants, these individuals had available CSF data and were grouped according to their clinical diagnoses and phosphorylated tau (T) status (n = 1182). According to a previous standard, we set the cutoff value at 27 pg/mL for p-tau to select participants with fibrillar tau (> 27 pg/mL; T+). Categorical and continuous measures are presented as numbers (%) or means ± standard deviations. **CADs** include patients who have (or have not) undergone stent placement or bypass grafting. **CDs** refer to transient ischemic attack and ischemic stroke. **Logical memory** indicates the total number of story units that are recalled. **ADNI_MEM/EF/LAN/VS** refers to the composite measures of memory function/executive function/language function/visuospatial function derived from the ADNI database. **MetaROI** includes the left angular gyrus, right angular gyrus, bilateral posterior cingulate gyrus, left inferior temporal gyrus, and right inferior temporal gyrus, which are the most important hypometabolic regions indicative of pathological metabolic changes in patients with AD. **B_HP volume** indicates the total volume of bilateral hippocampus. **GM volume** indicates the cerebral gray matter volume. **Ent_thickness** indicates the average thickness of bilateral entorhinal cortices. **WMH volume (Logarithmization)** means natural log-transformed WMH (a) or log-transformed total intracranial volume-normalized WMH (b).

Abbreviations: NC, cognitively normal control; MCI, mild cognitive impairment; APOE, apolipoprotein E; CADs, Coronary artery diseases; CDs, Cerebrovascular diseases; MMSE, Mini-Mental State Examination; MoCA, Montreal Cognitive Assessment; ADAS-Cog 13, Alzheimer's Disease Assessment Scale-Cognitive 13; ADNI, Alzheimer's Disease Neuroimaging Initiative; MEM, memory sub-domain; EF, executive function sub-domain; LAN, language sub-domain; VSP, visuospatial sub-domain; CSF, cerebrospinal fluid; Aβ, β-amyloid; p-tau, phosphorylated tau; t-tau, total tau; GAP43, growth associated protein-43; FDG, [^{18}F]fluoro-2-deoxyglucose; ROI, region of interest; SUVR, standardized uptake value ratio; B, bilateral; HP, hippocampus; WMH, white matter hyperintensity; GM, gray matter; Ent, entorhinal cortex; AD, Alzheimer's disease.

Supplementary Table 4. Baseline participant characteristics

	A-T- NC (N = 263)	A-T+ NC (N = 54)	A+T- NC (N = 109)	A+T+ NC (N = 48)	A-T- MCI (N = 203)	A-T+ MCI (N = 44)	A+T- MCI (N = 145)	A+T+ MCI (N = 150)	A-T- dementia (N = 10)	A-T+ dementia (N = 7)	A+T- dementia (N = 40)	A+T+ dementia (N = 109)
**age at baseline**	71.2 ± 6.22	75.3 ± 6.95	71.9 ± 6.22	75.9 ± 6.37	69.6 ± 7.55	72.8 ± 8.14	71.9 ± 7.40	74.0 ± 6.62	76.6 ± 9.68	79.1 ±2.59	74.2 ± 7.86	73.5 ± 8.67
**male**	109 (41.4%)	22 (40.7%)	47 (43.1%)	19 (39.6%)	102 (50.2%)	23 (52.3%)	97 (66.9%)	81 (54.0%)	9 (90.0%)	5 (71.4%)	29 (72.5%)	58 (53.2%)
**education**	16.9 ± 2.37	17.0 ± 2.47	16.7 ± 2.20	16.3 ± 2.34	16.2 ± 2.57	16.3 ± 2.16	16.4 ± 2.73	16.0 ± 2.67	16.8 ± 1.99	16.1 ± 2.55	16.6 ± 2.90	15.4 ± 2.50
***APOE* ε4-/-**	210 (80.2%, 262Ava)	35 (64.8%)	55 (52.4%, 105Ava)	19 (39.6%)	156 (80.0%, 195Ava)	27 (61.4%)	59 (41.8%, 141Ava)	37 (25.3%, 146Ava)	9 (90.0%)	2 (28.6%)	10 (25.0%)	31 (28.7%, 108Ava)
***APOE* ε4+/-**	50 (19.1%, 262Ava)	19 (35.2%)	42 (40.0%, 105Ava)	24 (50.0%)	37 (19.0%, 195Ava)	16 (36.4%)	57 (40.4%, 141Ava)	78 (53.4%, 146Ava)	1 (10.0%)	4 (57.1%)	22 (55.0%)	53 (49.1%, 108Ava)
***APOE* ε4+/+**	2 (0.8%, 262Ava)	0 (0%)	8 (7.6%, 105Ava)	5 (10.4%)	2 (1.0%, 195Ava)	1 (2.3%)	25 (17.7%, 141Ava)	31 (21.2%, 146Ava)	0 (0%)	1 (14.3%)	8 (20.0%)	24 (22.2%, 108Ava)
**Past medical histories**	n = 262	n = 54	n = 109	n = 47	n = 203	n = 44	n = 145	n = 150	n = 10	n = 7	n = 40	n = 108
**Hypertension**	111 (42.4%)	23 (42.6%)	54 (49.5%)	22 (46.8%)	106 (52.2%)	19 (43.2%)	74 (51.0%)	69 (46.0%)	8 (80.0%)	3 (42.9%)	20 (50.0%)	47 (43.5%)
**Diabetes**	31 (11.8%)	10 (18.5%)	10 (9.2%)	3 (6.4%)	27 (13.3%)	6 (13.6%)	15 (10.3%)	14 (9.3%)	4 (40.0%)	1 (14.3%)	6 (15.0%)	12 (11.1%)
**Hyperlipidemia**	132 (50.4%)	34 (63.0%)	51 (46.8%)	32 (68.1%)	103 (50.7%)	27 (61.4%)	80 (55.2%)	79 (52.7%)	3 (30.0%)	5 (71.4%)	25 (62.5%)	57 (52.8%)
**Smoking**	72 (27.5%)	14 (25.9%)	27 (24.8%)	15 (31.9%)	66 (32.5%)	8 (18.2%)	54 (37.2%)	53 (35.3%)	5 (50.0%)	3 (42.9%)	12 (30.0%)	37 (34.3%)
**Atrial fibrillation**	12 (4.6%)	0 (0%)	3 (2.8%)	4 (8.5%)	4 (2.0%)	1 (2.3%)	8 (5.5%)	4 (2.7%)	2 (20.0%)	0 (0%)	2 (5.0%)	1 (0.9%)
**CADs**	22 (8.4%)	1 (1.9%)	12 (11.0%)	8 (17.0%)	20 (9.9%)	6 (13.6%)	14 (9.7%)	21 (14.0%)	2 (20.0%)	1 (14.3%)	6 (15.0%)	11 (10.2%)
**CDs**	11 (4.2%)	2 (3.7%)	4 (3.7%)	1 (2.1%)	9 (4.4%)	0 (0%)	6 (4.1%)	7 (4.7%)	1 (10.0%)	0 (0%)	0 (0%)	4 (3.7%)
**Neuropsychological scales**												
**MMSE**	29.2 ± 1.08	29.2 ± 1.28	29.0 ± 1.17	29.0 ± 1.21	28.5 ± 1.59	28.2 ± 1.59	28.2 ± 1.70	27.2 ± 2.05	24.3 ± 1.64	23.6 ± 1.72	23.3 ± 2.12	22.7 ± 2.40
**MoCA**	26.2 ± 2.47 (262Ava)	26.0 ± 2.67 (53Ava)	25.6 ± 2.56 (108Ava)	24.8 ± 2.77	24.2 ± 3.00	23.2 ± 2.65 (42Ava)	23.2 ± 2.80 (144Ava)	21.7 ± 3.31	19.1 ± 4.33	19.0 ± 4.24	17.6 ± 4.64	16.3 ± 4.73 (108Ava)
**ADAS-cog13**	8.3 ± 4.41 (260Ava)	8.4 ± 3.53	8.8 ± 4.63	9.5 ± 4.60	11.7 ± 5.17 (202Ava)	15.9 ± 6.62 (43Ava)	15.2 ± 5.73 (144Ava)	18.7 ± 7.04	26.6 ± 6.02	24.9 ± 4.91	28.4 ± 7.56 (38Ava)	33.0 ± 8.56 (107Ava)
**Logical Memory**	13.7 ± 3.37	13.9 ± 3.07	12.9 ± 2.95	13.2 ± 2.98	8.4 ± 2.48	6.8 ± 3.68 (42Ava)	7.1 ± 3.24	5.3 ± 3.41	1.8 ± 2.78	2.6 ± 2.82	1.7 ± 2.04	1.5 ± 1.72 (108Ava)
**ADNI_MEM**	1.12 ± 0.584	1.05 ± 0.499	1.01 ± 0.604	0.88 ± 0.588	0.71 ± 0.590	0.22 ± 0.616	0.26 ± 0.529	-0.05 ± 0.602	-0.58 ± 0.370	-0.63 ± 0.464	-0.80 ± 0.515	-0.94 ± 0.519

**ADNI_EF**	1.12 ± 0.797	1.07 ± 0.932	0.83 ± 0.805	0.70 ± 0.811	0.70 ± 0.812	0.49 ± 0.794	0.32 ± 0.848 (144Ava)	-0.01 ± 0.836	-0.13 ± 0.943	-0.59 ± 0.985	-0.90 ± 1.170 (39Ava)	-0.93 ± 0.970 (107Ava)
**ADNI_LAN**	0.96 ± 0.705	0.94 ± 0.793	0.88 ± 0.708	0.71 ± 0.697	0.58 ± 0.698	0.31 ± 0.564	0.35 ± 0.800	0.04 ± 0.725	-0.45 ± 0.739	-0.36 ± 0.829	-0.51 ± 0.877	-0.85 ± 0.938
**ADNI_VS**	0.22 ± 0.636	0.19 ± 0.582	0.17 ± 0.623	0.09 ± 0.710	0.10 ± 0.686	-0.03 ± 0.712	-0.002 ± 0.748	-0.19 ± 0.764	-0.62 ± 0.722	-0.60 ± 1.323	-0.58 ± 1.016	-0.65 ± 1.087
**CSF core biomarkers**												
**$A\beta_{42}$ (pg/mL)**	1650.6 ± 481.63	1852.6 ± 779.34	727.1 ± 184.24	696.2 ± 154.25	1595.1 ± 436.01	1616.5 ± 671.59	654.6 ± 180.54	668.8 ± 163.14	1486.6 ± 415.90	1822.6 ± 920.81	544.3 ± 185.00	608.6 ± 158.18
**p-tau (pg/mL)**	17.96 ± 4.312	34.00 ± 7.487	16.38 ± 5.330	38.15 ± 9.047	17.68 ± 4.602	37.22 ± 14.016	18.40 ± 5.243	42.70 ± 14.248	18.71 ± 5.500	45.22 ± 16.879	21.94 ± 3.996	43.11 ± 13.937
**t-tau (pg/mL)**	206.97 ± 47.679	374.75 ± 72.869	180.51 ± 52.606	370.25 ± 67.667	203.41 ± 50.707	388.29 ± 113.933	197.47 ± 51.419	411.91 ± 123.175	229.62 ± 67.817	495.31 ± 168.138	232.09 ± 42.626	426.09 ± 133.011
**CSF GAP43 (pg/mL)**	4554.2 ± 2020.50 (134Ava)	7706.5 ± 3055.22 (32Ava)	3433.3 ± 1494.76 (58Ava)	7631.5 ± 3356.48 (24Ava)	4007.8 ± 1663.67 (155Ava)	7632.6 ± 3047.59 (32Ava)	3499.4 ± 1316.66 (105Ava)	7312.6 ± 3127.47 (120Ava)	6102.0 ± 2662.21 (8Ava)	8725.0 ± 3060.91 (6Ava)	3891.7 ± 1701.57 (33Ava)	7161.2 ± 3103.48 (79Ava)
**Neuroimaging**												
**FDG (metaROI SUVR)**	1.31 ± 0.054 (143Ava)	1.28 ± 0.046 (34Ava)	1.29 ± 0.046 (61Ava)	1.30 ± 0.070 (27Ava)	1.29 ± 0.064 (198Ava)	1.26 ± 0.065 (42Ava)	1.26 ± 0.075 (140Ava)	1.24 ± 0.079 (148Ava)	1.25 ± 0.079 (10Ava)	1.21 ± 0.072 (7Ava)	1.13 ± 0.128 (38Ava)	1.12 ± 0.112 (106Ava)
**B_HP volume (cm^3)**	6.74 ± 0.780 (263Ava)	6.60 ± 0.734 (54Ava)	6.70 ± 0.718 (109Ava)	6.55 ± 0.606 (48Ava)	6.58 ± 0.911 (203Ava)	6.22 ± 0.919 (44Ava)	6.44 ± 0.832 (145Ava)	6.05 ± 0.870 (150Ava)	5.13 ± 1.095 (10Ava)	4.81 ± 0.540 (7Ava)	5.87 ± 0.747 (40Ava)	5.43 ± 0.903 (109Ava)
**GM volume (cm^3)**	510.85 ± 50.171 (263Ava)	510.80 ± 53.768 (54Ava)	501.43 ± 49.176 (109Ava)	509.19 ± 39.222 (48Ava)	501.12 ± 48.375 (203Ava)	502.64 ± 45.735 (44Ava)	505.58 ± 48.861 (145Ava)	489.32 ± 51.676 (150Ava)	497.98 ± 43.129 (10Ava)	462.55 ± 37.651 (7Ava)	502.48 ± 49.166 (40Ava)	472.64 ± 57.561 (109Ava)
**Ent_thickness (mm)**	3.61 ± 0.283 (263Ava)	3.53 ± 0.34 (54Ava)	3.58 ± 0.295 (109Ava)	3.56 ± 0.259 (48Ava)	3.49 ± 0.435 (203Ava)	3.25 ± 0.564 (44Ava)	3.37 ± 0.445 (145Ava)	3.2 ± 0.442 (150Ava)	2.91 ± 0.628 (10Ava)	2.55 ± 0.593 (7Ava)	2.94 ± 0.512 (40Ava)	2.72 ± 0.464 (109Ava)
**WMH volume (cm^3)**	3.53 ± 4.724 (263Ava)	4.35 ± 5.254 (54Ava)	5.74 ± 7.532 (109Ava)	9.53 ± 20.085 (48Ava)	4.81 ± 7.351 (203Ava)	6.20 ± 7.183 (44Ava)	7.35 ± 9.274 (145Ava)	7.54 ± 9.189 (150Ava)	6.00 ± 8.107 (10Ava)	2.98 ± 1.266 (7Ava)	10.11 ± 11.854 (40Ava)	7.64 ± 9.046 (109Ava)
**WMH volume (Logarithmization)[a]**	0.24 ± 0.578 (263Ava)	0.31 ± 0.587 (54Ava)	0.43 ± 0.588 (109Ava)	0.55 ± 0.567 (48Ava)	0.34 ± 0.555 (203Ava)	0.48 ± 0.654 (44Ava)	0.56 ± 0.584 (145Ava)	0.65 ± 0.473 (150Ava)	0.48 ± 0.538 (10Ava)	0.45 ± 0.165 (7Ava)	0.78 ± 0.475 (40Ava)	0.63 ± 0.517 (109Ava)
**WMH volume (Logarithmization)[b]**	-2.91 ± 0.577 (263Ava)	-2.84 ± 0.584 (54Ava)	-2.71 ± 0.585 (109Ava)	-2.60 ± 0.565 (48Ava)	-2.80 ± 0.555 (203Ava)	-2.67 ± 0.655 (44Ava)	-2.60 ± 0.577 (145Ava)	-2.50 ± 0.470 (150Ava)	-2.68 ± 0.545 (10Ava)	-2.69 ± 0.158 (7Ava)	-2.39 ± 0.470 (40Ava)	-2.52 ± 0.506 (109Ava)

The analysis was performed in a subset of the included 1803 participants, these individuals had available CSF data and were grouped according to their clinical diagnoses, amyloid status and phosphorylated tau (T) status (n = 1182). According to a previous standard, we set the cutoff value at 977 pg/mL for Aβ and 27 pg/mL for p-tau to select participants with Aβ deposition (< 977 pg/mL; A+) and fibrillar tau (> 27 pg/mL; T+). Categorical and continuous measures are presented as numbers (%) or means ± standard deviations. **CADs** include patients who have (or have not) undergone stent placement or bypass grafting. **CDs** refer to transient ischemic attack and ischemic stroke. **Logical memory** indicates the total number of story units that are recalled. **ADNI_MEM/EF/LAN/VS** refers to the composite measures of memory function/executive function/language function/visuospatial function derived from the ADNI database. **MetaROI** includes the left angular gyrus, right angular gyrus, bilateral posterior cingulate gyrus, left inferior temporal gyrus, and right inferior temporal gyrus, which are the most important hypometabolic regions indicative of pathological metabolic changes in patients with AD. **B_HP volume** indicates the total volume of bilateral hippocampus. **GM volume** indicates the cerebral gray matter volume. **Ent_thickness** indicates the average thickness of bilateral entorhinal cortices. **WMH volume (Logarithmization)** means natural log-transformed WMH (a) or log-transformed total intracranial volume-normalized WMH (b).

Abbreviations: NC, cognitively normal control; MCI, mild cognitive impairment; APOE, apolipoprotein E; CADs, Coronary artery diseases; CDs, Cerebrovascular diseases; MMSE, Mini-Mental State Examination; MoCA, Montreal Cognitive Assessment; ADAS-Cog 13, Alzheimer's Disease Assessment Scale-Cognitive 13; ADNI, Alzheimer's Disease Neuroimaging Initiative; MEM, memory sub-domain; EF, executive function sub-domain; LAN, language sub-domain; VSP, visuospatial sub-domain; CSF, cerebrospinal fluid; Aβ, β-amyloid; p-tau, phosphorylated tau; t-tau, total tau; GAP43, growth associated protein-43; FDG, [^{18}F]fluoro-2-deoxyglucose; ROI, region of interest; SUVR, standardized uptake value ratio; B, bilateral; HP, hippocampus; GM, gray matter; Ent, entorhinal cortex; WMH, white matter hyperintensity; AD, Alzheimer's disease.

Supplementary Table 5. Baseline participant characteristics

	NC (N = 493)		MCI (N = 563)	
	Mean ± SD (or %)	n	Mean ± SD (or %)	n
age at baseline	73.0 ± 6.76	493	72.5 ± 7.81	563
male	209 (42.4%)	493	322 (57.2%)	563
education	16.6 ± 2.50	493	16.1 ± 2.70	563
APOE ε4-/-	338 (68.6%)	493	296 (52.6%)	563
APOE ε4+/-	142 (28.8%)	493	210 (37.3%)	563
APOE ε4+/+	13 (2.6%)	493	57 (10.1%)	563
**Past medical histories**				
Hypertension	232 (47.2%)	492	281 (49.9%)	563
Diabetes	60 (12.2%)	492	69 (12.3%)	563
Hyperlipidemia	252 (51.2%)	492	309 (54.9%)	563
Smoking	147 (29.9%)	492	196 (34.8%)	563
Atrial fibrillation	25 (5.1%)	492	30 (5.3%)	563
Coronary artery diseases	50 (10.2%)	492	68 (12.1%)	563
Cerebrovascular diseases	24 (4.9%)	492	28 (5.0%)	563
**Neuropsychological scales**				
MMSE	29.04 ± 1.192	493	28.00 ± 1.793	563
MoCA	25.72 ± 2.644	490	23.16 ± 3.233	562
ADAS-cog13	8.74 ± 4.421	490	14.97 ± 6.633	560
Logical Memory	13.25 ± 3.342	493	7.13 ± 3.344	562
ADNI_MEM	1.05 ± 0.594	493	0.34 ± 0.674	563
ADNI_EF	0.94 ± 0.820	493	0.37 ± 0.883	563
ADNI_LAN	0.84 ± 0.712	493	0.34 ± 0.773	563
ADNI_VS	0.19 ± 0.634	493	-0.02 ± 0.744	563
**CSF core biomarkers**				
$A\beta_{42}$ (pg/mL)	1354.16 ± 662.193	391	1067.36 ± 565.004	485
p-tau (pg/mL)	21.64 ± 9.452	391	26.46 ± 14.724	485
t-tau (pg/mL)	237.93 ± 91.014	391	274.95 ± 129.263	485
**CSF GAP43 (pg/mL)**	4952.72 ± 2712.091	235	5149.99 ± 2858.624	396
**Neuroimaging**				
FDG (metaROI SUVR)	1.30 ± 0.054	295	1.27 ± 0.070	541
B_HP volume (cm^3)	6.67 ± 0.754	493	6.37 ± 0.913	563
GM volume (cm^3)	506.11 ± 48.237	493	498.39 ± 48.751	563
Ent_thickness (mm)	3.55 ± 0.291	493	3.34 ± 0.466	563
WMH volume (cm^3)	5.40 ± 9.283	493	6.70 ± 8.743	563
WMH volume (Logarithmization)[a]	0.38 ± 0.584	493	0.52 ± 0.566	563
WMH volume (Logarithmization)[b]	-2.77 ± 0.582	493	-2.62 ± 0.563	563
**Participants with follow-up WMH/ADNI_MEM/ADNI_EF data within the next 48 months**				
N at baseline	493/493/493		563/563/563	
N at 12 months	283/300/300		520/539/538	
N at 24 months	361/403/403		406/446/443	
N at 36 months	43/82/79		139/374/369	
N at 48 months	161/246/244		198/291/283	

The analysis was performed in a subset of the included 1803 participants, these individuals were non-dementia, had complete demographic information and received one or more clinical follow-up and WMH reexamination within the next 48 months (n = 1056). Categorical and continuous measures are presented as numbers (%) or means ± standard deviations. **Coronary artery diseases** include patients who have (or have not) undergone stent placement or bypass grafting. **Cerebrovascular diseases** refer to transient ischemic attack and ischemic stroke. **Logical memory** indicates the total number of story units that are recalled. **ADNI_MEM/EF/LAN/VS** refers to the composite measures of memory function/executive function/language function/visuospatial function derived from the ADNI database. **MetaROI** includes the left angular gyrus, right angular gyrus, bilateral posterior cingulate gyrus, left inferior temporal gyrus, and right inferior temporal gyrus, which are the most important hypometabolic regions indicative of pathological metabolic changes in patients with AD. **B_HP volume** indicates the total volume of bilateral hippocampus. **GM volume** indicates the cerebral gray matter volume. **Ent_thickness** indicates the average thickness of bilateral entorhinal cortices. **WMH volume (Logarithmization)** means natural log-transformed WMH (a) or log-transformed total intracranial volume-normalized WMH (b).

Abbreviations: NC, cognitively normal control; MCI, mild cognitive impairment; APOE, apolipoprotein E; MMSE, Mini-Mental State Examination; MoCA, Montreal Cognitive Assessment; ADAS-Cog 13, Alzheimer's Disease Assessment Scale-Cognitive 13; ADNI, Alzheimer's Disease Neuroimaging Initiative; MEM, memory sub-domain; EF, executive function sub-domain; LAN, language sub-domain; VSP, visuospatial sub-domain; CSF, cerebrospinal fluid; Aβ, β-amyloid; p-tau, phosphorylated tau; t-tau, total tau; GAP43, growth-associated protein-43; FDG, [^{18}F]fluoro-2-deoxyglucose; ROI, region of interest; SUVR, standardized uptake value ratio; B, bilateral; HP, hippocampus; GM, gray matter; Ent, entorhinal cortex; WMH, white matter hyperintensity; AD, Alzheimer's disease.

Supplementary Table 6. Relationships between brain WMH volume and AD CSF core biomarkers

	NC group (n = 467)				MCI group (n = 526)				Dementia group (n = 164)			
	**β**	**95% CI**	**SE**	***p***	**β**	**95% CI**	**SE**	***p***	**β**	**95% CI**	**SE**	***p***
**Intercept**	-4.4561	-5.1609 ~ -3.7513	0.3586	**< 0.0001**	-4.7160	-5.2886 ~ -4.1434	0.2914	**< 0.0001**	-4.1054	-4.9919 ~ -3.2190	0.4486	**< 0.0001**
**Age**	0.0263	0.0181 ~ 0.0346	0.0042	**< 0.0001**	0.0331	0.0270 ~ 0.0393	0.0031	**< 0.0001**	0.0233	0.0145 ~ 0.0321	0.0045	**< 0.0001**
**Female**	0.0826	-0.0194 ~ 0.1846	0.0519	0.1123	0.0285	-0.0605 ~ 0.1176	0.0453	0.5292	0.0345	-0.1310 ~ 0.2000	0.0838	0.6810
**Education levels**	-0.0103	-0.0314 ~ 0.0109	0.0107	0.3400	-0.0181	-0.0348 ~ -0.0015	0.0085	**0.0332**	0.0012	-0.0272 ~ 0.0296	0.0144	0.9335
***APOE* ε4+/-**	-0.0846	-0.2002 ~ 0.0311	0.0588	0.1514	-0.0650	-0.1663 ~ 0.0363	0.0516	0.2082	-0.0254	-0.1923 ~ 0.1415	0.0844	0.7640
***APOE* ε4+/+**	0.0051	-0.2803 ~ 0.2905	0.1452	0.9721	0.0660	-0.0945 ~ 0.2265	0.0817	0.4197	-0.1510	-0.3677 ~ 0.0657	0.1097	0.1706
**Hypertension**	0.1717	0.0697 ~ 0.2736	0.0519	**0.0010**	0.0961	0.0085 ~ 0.1837	0.0446	**0.0316**	0.0040	-0.1463 ~ 0.1542	0.076	0.9585
**Diabetes**	-0.0067	-0.1622 ~ 0.1487	0.0791	0.9321	0.1191	-0.0207 ~ 0.2588	0.0711	0.0948	-0.1355	-0.3450 ~ 0.0741	0.106	0.2035
**Atrial fibrillation**	0.0489	-0.2003 ~ 0.2981	0.1268	0.7000	0.0528	-0.1860 ~ 0.2915	0.1215	0.6644	0.3061	-0.1064 ~ 0.7186	0.2087	0.1446
**Smoking**	0.1615	0.0523 ~ 0.2708	0.0556	**0.0038**	0.0688	-0.0201 ~ 0.1577	0.0453	0.1289	0.1055	-0.0470 ~ 0.2579	0.0772	0.1738
**Hyperlipidemia**	-0.0501	-0.1515 ~ 0.0514	0.0516	0.3328	0.0350	-0.0515 ~ 0.1215	0.0440	0.4268	0.1094	-0.0380 ~ 0.2568	0.0746	0.1447
**CADs**	-0.0154	-0.1900 ~ 0.1592	0.0888	0.8627	0.0226	-0.1170 ~ 0.1623	0.0711	0.7502	-0.1145	-0.3393 ~ 0.1104	0.1138	0.3161
**CDs**	0.2630	0.0122 ~ 0.5138	0.1276	**0.0399**	-0.0263	-0.2494 ~ 0.1968	0.1136	0.8170	0.1996	-0.2219 ~ 0.6212	0.2133	0.3509
**CSF Aβ_{42} levels**	-0.0002	-0.0003 ~ -0.0001	< 0.0001	**< 0.0001**	-0.0001	-0.0002 ~ -0.0001	< 0.0001	**0.0023**	-0.0003	-0.0004 ~ -0.0001	0.0001	**0.0031**
**CSF p-tau levels**	0.0019	-0.0036 ~ 0.0074	0.0028	0.4920	0.0008	-0.0024 ~ 0.0040	0.0016	0.6261	-0.0002	-0.0049 ~ 0.0044	0.0024	0.9185

The analysis was performed in a subset of the included 1803 participants, these individuals had complete clinical information and available CSF data (n = 1157; n = 467 for NC group, n = 526 for MCI group, and n = 164 for dementia group); compared with the participants in **Table S2-S4**, 25 participants were excluded due to incomplete clinical information. CSF core biomarkers, including Aβ_{42} and p-tau levels, plus age, sex, *APOE* ε4 status, comorbidities statuses, and TIV were used as predictors of brain WMH volumes ((total intracranial volume-normalized and log-transformed). CSF t-tau was not included due to its extremely high correlation with p-tau ($R > 0.900$, $p < 0.001$).

Abbreviations: CI, confidence interval; SE, standard error; WMH, white matter hyperintensity; AD, Alzheimer's disease; CSF, cerebrospinal fluid; MCI, mild cognitive impairment; APOE, apolipoprotein E; CADs, Coronary artery diseases; CDs, Cerebrovascular diseases; Aβ, β-amyloid; p-tau, phosphorylated tau; t-tau, total tau.

Supplementary Table 7. Cox regression analysis for dementia conversion from 876 non-dementia participants

	HR	95% CI	*p*
**Model 1**			
WMH volume	1.013	0.999 ~ 1.026	0.0621
**Model 2**			
WMH volume	1.007	0.993 ~ 1.022	0.3201
**Model 3**			
WMH volume	1.002	0.986 ~ 1.017	0.8395
CSF Aβ_{42} levels	0.998	0.998 ~ 0.999	**< 0.0001**
CSF p-tau levels	1.038	1.028 ~ 1.049	**< 0.0001**

Eight hundred and seventy-six non-dementia participants (see **Table 1**), with complete data including demography information, *APOE* data, and AD CSF core biomarkers, were followed up 6-48 months. Among them, 119 participants developed to dementia during the follow-up period. The analysis was performed using Cox regression method. In model 1, WMH volume and TIV were used as predictors of outcome events; in model 2, WMH volume, TIV, plus age, sex, and *APOE* ε4 status were used as predictors of outcome events; in model 3, WMH volume, TIV, age, sex, *APOE* ε4 status, plus CSF Aβ_{42} and CSF p-tau levels were used as predictors of outcome events. CSF t-tau was not included due to its extremely high correlation with p-tau ($R > 0.900$, $p < 0.001$).

Abbreviations: HR, hazard ratio; CI, confidence interval; APOE, apolipoprotein E; WMH, white matter hyperintensity; TIV, total intracranial volume; CSF, cerebrospinal fluid; Aβ, β-amyloid; p-tau, phosphorylated tau; t-tau, total tau.

Supplementary Table 8. The proportional hazards assumption was tested using the scaled *Schoenfeld* residuals

	chisq	***p***
**Model 1**		
WMH volume	0.511	0.472
TIV	0.428	0.513
Global	0.990	0.611
**Model 2**		
WMH volume	0.4015	0.526
TIV	0.2236	0.636
Age	3.9493	0.053
Sex	0.0145	0.904
APOE ε4 status	4.2621	0.119
Global	7.4461	0.282
**Model 3**		
WMH volume	0.502	0.479
TIV	0.202	0.653
Age	4.760	**0.039**
Sex	0.000952	0.975
APOE ε4 status	3.071	0.215
CSF Aβ_{42} levels	1.020	0.312
CSF p-tau levels	1.284	0.259
Global	0.103	0.246

In Model 3, the covariate age does not meet the assumption of proportional hazards ($p < 0.05$). Adding the interaction term of age and time as an additional covariate had no effect on the Cox regression analysis results (**data not shown**).

Abbreviations: APOE, apolipoprotein E; WMH, white matter hyperintensity; TIV, total intracranial volume; CSF, cerebrospinal fluid; Aβ, β-amyloid; p-tau, phosphorylated tau.

Supplementary Table 9. Relationships between baseline brain WMH volume and cognitive function slopes

	ΔMemory			ΔEF		
	**β**	**95% CI**	***p***	**β**	**95% CI**	***p***
**Model 1**						
MCI	-0.0045	-0.0057 ~ -0.0034	**< 0.0001**	-0.0016	-0.0026 ~ -0.0005	**0.0028**
**WMH**	-0.0019	-0.0030 ~ -0.0008	**0.0004**	-0.0009	-0.0019 ~ 0.0001	0.0671
**Model 2**						
MCI	-0.0045	-0.0057 ~ -0.0034	**< 0.0001**	-0.0016	-0.0026 ~ -0.0005	**0.0028**
**WMH**	-0.0018	-0.0029 ~ -0.0007	**0.0011**	-0.0009	-0.0019 ~ 0.0001	0.0685
**Model 3**						
MCI	-0.0035	-0.0048 ~ -0.0023	**< 0.0001**	-0.0005	-0.0016 ~ 0.0007	0.4404
**WMH**	-0.0014	-0.0025 ~ -0.0001	**0.0466**	-0.0005	-0.0016 ~ 0.0006	0.3818
CSF Aβ_{42}	0.000003	0.000002 ~ 0.000006	**< 0.0001**	0.000003	0.000002 ~ 0.000004	**< 0.0001**
CSF p-tau	-0.0002	-0.0003 ~ -0.0002	**< 0.0001**	-0.0002	-0.0002 ~ -0.0001	**< 0.0001**
**Model 4**						
MCI	-0.0035	-0.0048 ~ -0.0023	**< 0.0001**	-0.0005	-0.0016 ~ 0.0007	0.4419
**WMH**	-0.0019	-0.0042 ~ 0.0004	0.1053	0.0002	-0.0019 ~ 0.0023	0.8308
CSF Aβ_{42}	0.000005	0.00000002 ~ 0.00000900	**0.0481**	0.000001	-0.000003 ~ 0.000005	0.5654
WMH * Aβ_{42}	0.0000004	-0.0000012 ~ 0.0000020	0.6105	-0.0000006	-0.0000021 ~ 0.0000009	0.4348
CSF p-tau	-0.0002	-0.0003 ~ -0.0002	**< 0.0001**	-0.0002	-0.0002 ~ -0.0001	**< 0.0001**

The cognitive function slopes were calculated by using linear mixed-effects models among non-dementia participants with at least one follow-up ADNI_MEM/EF score within the next 48 months (n = 1056, see **Table S5**). In model 1, baseline WMH volume, plus age, sex, education, *APOE* ε4 status, and cognitive status were used as predictors of cognitive function slopes. In model 2, hypertension and smoking status were used as additional predictors on the basis of model 1. In model 3, CSF core biomarkers, including Aβ_{42} and p-tau levels, were used as additional predictors on the basis of model 2. In model 4, the interaction term of CSF Aβ_{42} and WMH were used as additional predictors on the basis of model 3. The WMH volume was total intracranial volume-normalized and log-transformed. CSF t-tau was not included due to its extremely high correlation with p-tau ($R > 0.900$, $p < 0.001$).

Abbreviations: MCI, mild cognitive impairment; WMH, white matter hyperintensity; CSF, cerebrospinal fluid; Aβ, β-amyloid; p-tau, phosphorylated tau; t-tau, total tau; APOE, apolipoprotein E; MEM, memory sub-domain; EF, executive function; ADNI, Alzheimer's Disease Neuroimaging Initiative; CI, confidence interval.

Supplementary Table 10. Change in WMH volume within the next 48 months in different cognitive groups

	Total individuals		
	**β**	**SE**	***p***
**Baseline WMH**	0.9936	0.0037	**< 0.0001**
**MCI**	0.0074	0.0782	0.9246
**Dementia**	0.0150	0.1312	0.9088
**Time**	0.0440	0.0065	**< 0.0001**
**MCI * Time**	0.0030	0.0088	0.7319
**Dementia * Time**	0.0343	0.0162	**0.0340**

The results were obtained from a linear mixed-effects model adjusted for age, sex, educational level, *APOE* ε4 status, total intracranial volume, and baseline WMH volume.

Abbreviations: SE, standard error; MCI, mild cognitive impairment; WMH, white matter hyperintensity; APOE, apolipoprotein E.

Supplementary Table 11. Change in WMH volume within the next 48 months in the groups with different amyloid status

	NC group			MCI group			Dementia group		
	**β**	**SE**	***p***	**β**	**SE**	***p***	**β**	**SE**	***p***
**Baseline WMH**	0.9921	0.0055	**< 0.0001**	0.9806	0.0066	**< 0.0001**	0.9866	0.0122	**< 0.0001**
**Aβ+ status**	-0.1118	0.1178	0.3429	-0.2133	0.1343	0.1125	-0.1250	0.3785	0.7420
**Time**	0.0362	0.0080	**< 0.0001**	0.0261	0.0078	**< 0.0001**	0.0824	0.0634	0.1960
**Aβ+ status * Time**	0.0282	0.0139	**0.0434**	0.0356	0.0106	**< 0.0001**	-0.0195	0.0674	0.7730

The results were obtained from a linear mixed-effects model adjusted for age, sex, educational level, *APOE* ε4 status, total intracranial volume, and baseline WMH volume.

Abbreviations: SE, standard error; MCI, mild cognitive impairment; WMH, white matter hyperintensity; APOE, apolipoprotein E.

Supplementary Table 12. Relationships between ΔWMH volume and cognitive function slopes

	β	95% CI	SE	*p*
**ΔMemory**				
MCI group	-0.0036	-0.0049 ~ -0.0024	**0.0006**	**< 0.001**
CSF Aβ42 levels	0.000003	0.0000023 ~ 0.0000044	**0.0000005**	**< 0.001**
CSF p-tau levels	-0.0002	-0.00027 ~ -0.00017	**0.000025**	**< 0.001**
ΔWMH volume	-0.0233	-0.0364 ~ -0.0102	**0.0067**	**< 0.001**
**ΔEF**				
MCI group	-0.00047	-0.00161 ~ 0.00067	0.00058	0.4206
CSF Aβ42 levels	0.0000028	0.0000018 ~ 0.0000037	0.00000049	**< 0.001**
CSF p-tau levels	-0.00017	-0.00021 ~ -0.00012	0.000023	**< 0.001**
ΔWMH volume	-0.0198	-0.0318 ~ -0.0078	**0.0061**	**0.0012**

The slopes (ΔWMH, ΔMemory and ΔEF) were calculated by using linear mixed-effects models among non-dementia participants with at least one follow-up WMH data, ADNI_MEM/EF score within the next 48 months (n = 1056, see **Table S5**). The ΔWMH volumes, plus diagnostic status, age, sex, education, *APOE* ε4 status, hypertension and smoking status, and CSF core biomarkers, including $A\beta_{42}$ and p-tau levels were used as predictors of cognitive function slopes (n = 876, see **Table 1**). CSF t-tau was not included due to its extremely high correlation with p-tau (R > 0.900, $p < 0.001$).

Abbreviations: MCI, mild cognitive impairment; WMH, white matter hyperintensity; CSF, cerebrospinal fluid; Aβ, β-amyloid; p-tau, phosphorylated tau; t-tau, total tau; APOE, apolipoprotein E; MEM, memory sub-domain; EF, executive function; ADNI, Alzheimer's Disease Neuroimaging Initiative; CI, confidence interval; SE, standard error.

Supplementary Table 13. Relationships between baseline brain WMH volume and entorhinal cortex thickness

	Entorhinal cortex thickness			ΔEntorhinal cortex thickness		
	β	95% CI	*p*	β	95% CI	*p*
**MCI**	-0.2125	-0.2646 ~ -0.1604	**< 0.0001**	-0.00720	-0.0117 ~ 0.00273	**0.00159**
**Dementia**	-0.7138	-0.7929 ~ -0.6346	**< 0.0001**	-0.00649	-0.0134 ~ 0.000436	0.06626
**CSF Aβ42 levels**	0.0000356	-0.000006919 ~ 0.00007822	**0.1006**	0.00000918	0.00000550 ~ 0.0000128	**< 0.0001**
**CSF p-tau levels**	-0.00276	-0.00462 ~ -0.000906	**0.00357**	-0.000479	-0.000637 ~ -0.000321	**< 0.0001**
**WMH volume**	-0.0577	-0.1045 ~ -0.01096	**0.01562**	-0.003431	-0.00748 ~ 0.000616	0.0955

Baseline WMH volume, plus diagnostic status, age, sex, education, *APOE* ε4 status, hypertension and smoking status, and CSF core biomarkers, including $A\beta_{42}$ and p-tau levels were used as predictors of cognitive function slopes. The WMH volume was total intracranial volume-normalized and log-transformed. CSF t-tau was not included due to its extremely high correlation with p-tau (R > 0.900, $p < 0.001$).

Abbreviations: MCI, mild cognitive impairment; WMH, white matter hyperintensity; CSF, cerebrospinal fluid; Aβ, β-amyloid; p-tau, phosphorylated tau; t-tau, total tau; APOE, apolipoprotein E; MEM, memory sub-domain; EF, executive function; ADNI, Alzheimer's Disease Neuroimaging Initiative; CI, confidence interval.

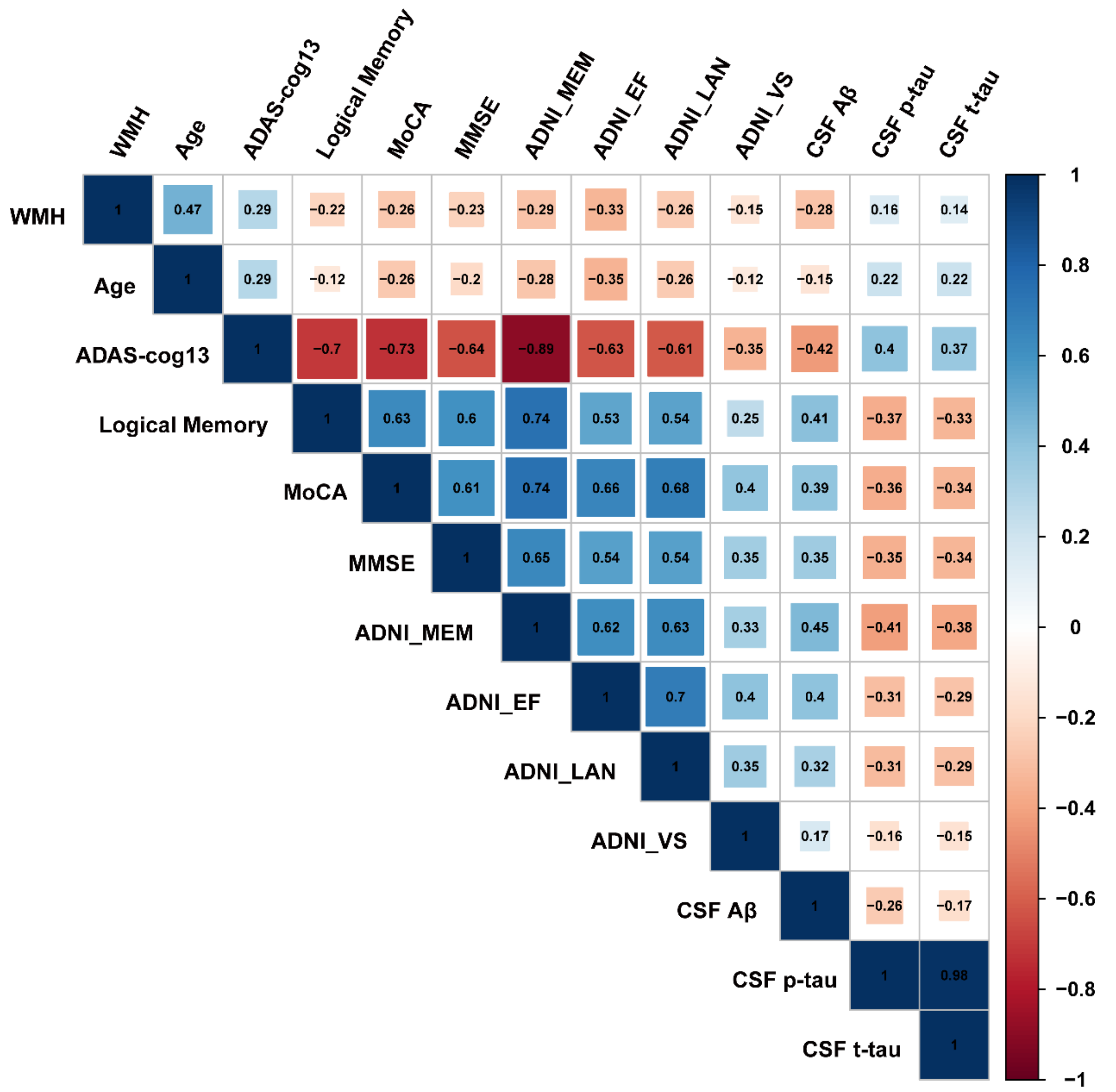

Supplementary Figure 1. Correlation analysis matrices

The matrix shows the spearman correlation coefficients between the WMH volume and age, scores of typical neuropsychology scales, and CSF biomarkers, including $A\beta_{42}$, p-tau and t-tau. **Logical memory** indicates the total number of story units that are recalled. The WMH volume was total intracranial volume-normalized and log-transformed.

Abbreviations: WMH, white matter hyperintensity; ADAS-Cog 13, Alzheimer's Disease Assessment Scale-Cognitive 13; MMSE, Mini-Mental State Examination; MoCA, Montreal Cognitive Assessment; ADNI, Alzheimer's Disease Neuroimaging Initiative; MEM, memory sub-domain; EF, executive function sub-domain; LAN, language sub-domain; VSP, visuospatial sub-domain; CSF, cerebrospinal fluid; Aβ, β-amyloid; p-tau, phosphorylated tau; t-tau, total tau.

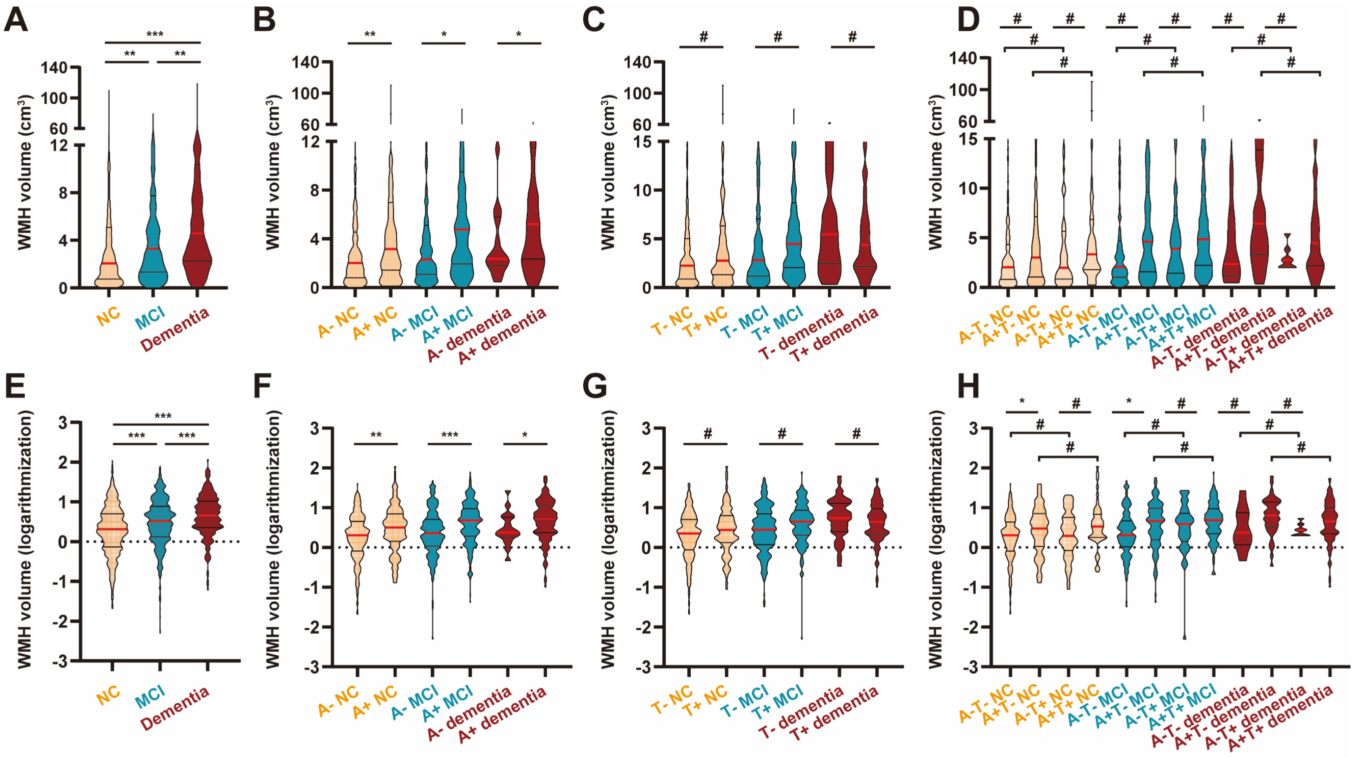

Supplementary Figure 2. WMH volume in different diagnostic groups

The WMH volume was raw (**A-D**) or log-transformed (**E-H**). (**A, E**) Participants were grouped by clinical diagnosis (n = 1803; see **Table S1**). (**B, F**) Participants were grouped by clinical diagnosis and CSF-determined Aβ status (n = 1182; see **Table S2**). (**C, G**) Participants were grouped by clinical diagnosis and CSF-determined p-tau status (n = 1182; see **Table S3**). (**D, H**) Participants were grouped by clinical diagnosis and CSF-determined Aβ and p-tau statuses (n = 1182; see **Table S4**). According to a previous standard, we set the cutoff value at 977 pg/mL for Aβ and 27 pg/mL for p-tau to select participants with Aβ deposition (< 977 pg/mL; A+) and fibrillar tau (> 27 pg/mL; T+). The results are presented in improved box charts; the red lines indicate the median values; the upper straight lines indicate the upper quartiles and the lower straight lines indicate the lower quartiles. Statistical analysis was conducted using one-way ANOVA followed by Tukey's test (adjusted *p* value). Comparisons among groups: *, < 0.05; **, < 0.01; ***, < 0.001; #, > 0.05.

Abbreviations: WMH, white matter hyperintensity; NC, cognitively normal control; MCI, mild cognitive impairment; CSF, cerebrospinal fluid; Aβ, β-amyloid; p-tau, phosphorylated tau; ANOVA, analysis of variance.

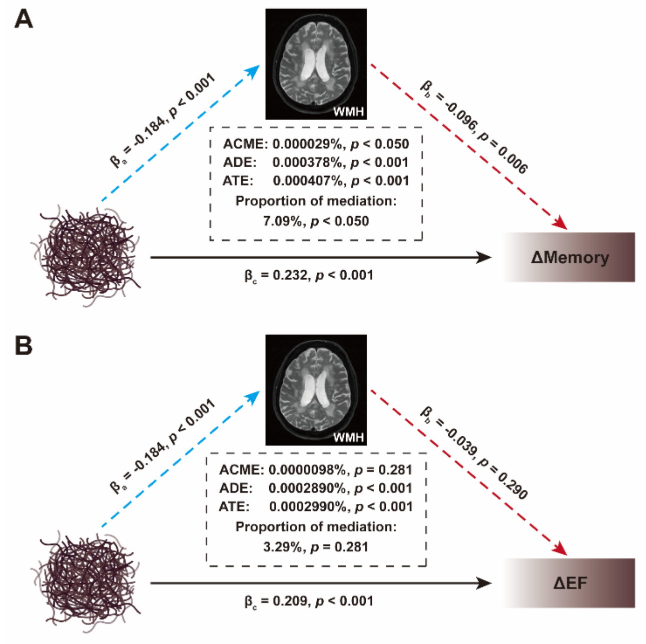

Supplementary Figure 3. Mediation analyses of brain Aβ deposition on cognitive function slopes
Baseline WMH volume was used as the mediating variable; age, sex, educational level, *APOE* ε4 status, and TIV were used as the covariates; the CSF Aβ levels as independent variable, and the ADNI_MEM slope or ADNI_EF slope as dependent variable. All paths are presented in standardized regression coefficients (β). The WMH volume was total intracranial volume-normalized and log-transformed. The analysis was performed in 876 participants in **Table 1**. Slopes (Δ) were calculated by using linear mixed-effects models among non-dementia participants with at least one follow-up ADNI_MEM/EF score within the next 48 months (n = 1056, see **Table S5**).
Abbreviations: WMH, white matter hyperintensity; CSF, cerebrospinal fluid; Aβ, β-amyloid; APOE, apolipoprotein E; TIV, total intracranial volume; MEM, memory sub-domain; EF, executive function; ADNI, Alzheimer’s Disease Neuroimaging Initiative; ACME, average causal mediation effect; ADE, average direct effect.

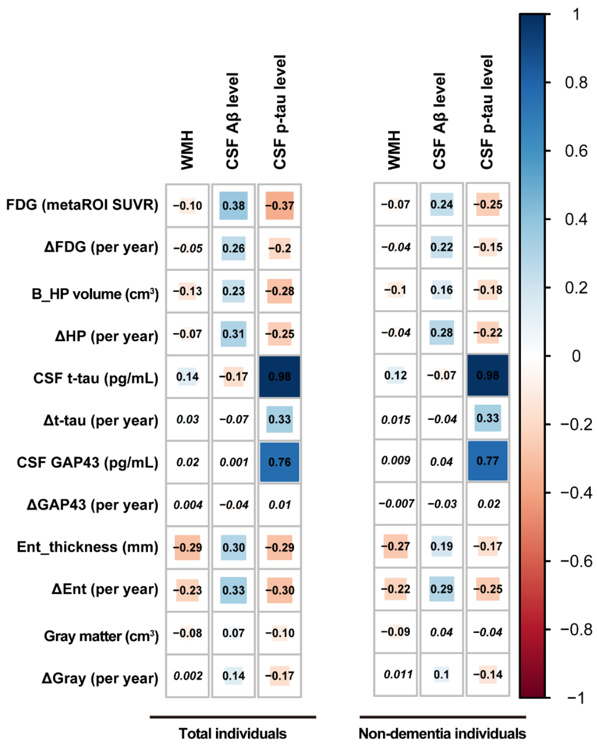

Supplementary Figure 4. Correlation analysis matrices

The analysis was performed among total participants or non-dementia participants; see **Table S2-S4** for clinical information. The matrix shows the spearman correlation coefficients between the WMH volumes or CSF Aβ_{42} levels or CSF p-tau levels and baseline levels or change slopes (Δ) of traditional neurodegeneration markers including FDG SUVR (metaROI), HP volumes (bilateral), CSF t-tau levels, CSF GAP43 levels, entorhinal cortex thickness (average), and cerebral gray matter volume. The presence of squares indicates significant ($p < 0.05$), while the italicized coefficients indicate no significant ($p > 0.05$). **MetaROI** includes the left angular gyrus, right angular gyrus, bilateral posterior cingulate gyrus, left inferior temporal gyrus, and right inferior temporal gyrus, which are the most important hypometabolic regions indicative of pathological metabolic changes in patients with AD. **B_HP volume** indicates the total bilateral volume. **Ent_thickness** indicates the average thickness of bilateral entorhinal cortices. **GM volume** indicates the cerebral gray matter volume. The WMH volume was total intracranial volume-normalized and log-transformed.

Abbreviations: WMH, white matter hyperintensity; CSF, cerebrospinal fluid; Aβ, β-amyloid; GAP43, growth-associated protein-43; FDG, [^{18}F]fluoro-2-deoxyglucose; ROI, region of interest; SUVR, standardized uptake value ratio; HP, hippocampus; t-tau, total tau; Ent, entorhinal cortex.
